# Transformative Moral Repair Following Interpersonal Transgressions: Post-Transgression Relationship Growth

**DOI:** 10.1177/01461672251358619

**Published:** 2025-08-11

**Authors:** Blake Quinney, Michael Wenzel, Tyler G. Okimoto, Michael Thai, Lydia Woodyatt

**Affiliations:** 1 The University of Adelaide, SA, Australia; 2 Flinders University, Adelaide, SA, Australia; 3 The University of Queensland, Brisbane, Australia

**Keywords:** interpersonal transgressions, stress-related growth, co-rumination, moral repair, justice, relationships, close relationships, communication, ethics/morality, forgiveness, interpersonal processes, interpersonal relationships, romantic relationships

## Abstract

Research often views relationship repair through a reparative lens of relationship partners attempting to fix what was damaged or broken by the transgression. We argue here for a transformative lens to view transgressions as potential catalysts for the strengthening of relationships or what we term: *post-transgression relationship growth* (PTRG). However, we also argue that PTRG is more likely achieved when the transgression is dealt with dyadically via a constructive process of co-reflection. Results from a pilot study and two pre-registered three-wave longitudinal studies provided validation for a PTRG scale, which assesses relationship growth/decline after transgressions that occurred between romantic relationship partners. Moreover, co-reflection was prospectively positively associated with PTRG when controlling for baseline relationship qualities. Together, these findings highlight that relationships can emerge stronger out of relationship adversity when relationship partners engage in the constructive process of co-reflection.

Relationship transgressions such as disrespect, deceit, and infidelity can damage the foundations romantic relationships are built on, such as shared values and trust (Okimoto & Wenzel, 2008). Psychological research typically emphasizes that damaged relationships can be mended by repairing what was lost or damaged in the transgression (e.g., trust; Lewicki & Brinsfield, 2017). However, could the process of relationship repair following transgressions in fact become a catalyst for relationship growth? Little attention has been paid to whether, and how, working through a wrongdoing may lead relationship partners to perceive themselves as having developed *new* relationship qualities or *strengthened* pre-existing ones (D. L. Kelley et al., 2018). We investigate whether transgressions represent opportunities for relationship transformation, what we term, *post-transgression relationship growth* (PTRG), and whether they do so specifically if dealt with via the constructive process of *co-reflection*—relationship partners working through the incident together (Thai et al., 2023). We present a multi-faceted PTRG scale for its measurement. Across a pilot study and two three-wave longitudinal studies, we conduct a psychometric evaluation and validation of the PTRG scale, and we investigate whether co-reflection promotes PTRG.

## Transformative Moral Repair: Beyond “Repairing” Broken Relationships

Interpersonal transgression research typically takes a reparative lens that individuals must repair or reinstate what was damaged in the wrongdoing. The reparative lens is evident in research on *moral repair*, the process of restoring or reconstructing the relationship’s ethics and moral relations (Wenzel et al., 2021). For example, moral repair research emphasizes that relationship partners must repair what was broken in the transgression (Woodyatt et al., 2022), often via each party working toward recouping post-transgression losses and addressing threats to the parties’ needs (Shnabel & Nadler, 2008). The reparative lens has a backward-looking view on what constitutes success for individuals overcoming transgressions. Relationship partners must work toward restoring the relationship that existed *before* the transgression occurred (Rusbult et al., 2007).

We advance moral repair theorizing to go beyond the reparative lens that individuals can only repair the damage to their relationships caused by transgressions. The reparative lens may not capture the full range of potential ways that relationships can develop or change in response to wrongdoing. In particular, it may overlook that, depending on how relationship partners respond to transgressions, relationships may in fact be changed, strengthened, or *transformed* (D. L. Kelley et al., 2018). We investigate whether interpersonal transgressions can be a catalyst for positive relationship transformation, or PTRG.

## Post-Transgression Relationship Growth

Our notion that relationships may experience growth after transgressions is theoretically inspired by the perceived growth literature (Jayawickreme et al., 2021). The literature contains different terminologies such as stress-related growth (Park et al., 1996), benefit finding (Davis et al., 1998), adversarial growth (Linley & Joseph, 2004), but the most notable term is post-traumatic growth (Tedeschi & Calhoun, 1996). Consistent across these different terminologies is the fundamental idea that individuals can experience growth in specific domains after stressful events. We argue that relationships, too, can experience growth after transgressions.

The literature on perceived growth is often limited in its focus on *personal* growth rather than *relationship* growth. For example, the *posttraumatic growth inventory* (Tedeschi & Calhoun, 1996) assesses domains such as personal strength. Although one of these domains refers to perceived growth in how one relates to others, that growth is *personal* (e.g., “a willingness to express my emotions”; Tedeschi & Calhoun, 1996) rather than a perception that the relationship has experienced growth (e.g., “we express our emotions to each other”). Despite relationship research highlighting that people in a relationship may view themselves as a collective or a couple identity—a “we” rather than two independent people (Cruwys et al., 2023; Emery et al., 2021)—relationship researchers have predominantly focused on how relationships are instrumental to personal growth (for similar critiques, see Feeney & Collins, 2015; Overall et al., 2010).

Specifically, we argue that overcoming serious transgressions that occur *within* romantic relationships may be the basis of relationship growth, a proposition that is supported by theory and research. For example, according to interdependence theory, transgressions are diagnostic situations for relationships because relationship partners may gain insight into each other’s values, and a sense of how the relationship would operate if it were to continue (Rusbult & Van Lange, 2003). In particular, if relationship partners engage in conciliatory behaviors to address transgressions, then this may offer insights that can strengthen relationships, such as that both partners value their relationship or want to be supportive toward the other. For example, in a cross-sectional study, Hannon et al. (2010, study 3) asked relationship partners to recall a recent transgression and how they resolved the incident. Conciliatory behaviors (e.g., offender making amends) were significantly related to betrayal resolution and, in turn, greater relationship quality (partly captured with relationship growth items such as “Our relationship is better now than ever before”). There is also qualitative evidence of relationship partners reporting relationship growth after severe transgressions. For example, D. Kelley (1998) examined relationship partners’ perceptions of their relationship after working through a transgression and forgiving their partner. It was found that 26% of relationship partners indicated strengthened relationships which was almost equal to the 29% who indicated deteriorated relationships. These findings suggest that relationship growth after transgressions is a relatively frequent phenomenon, yet underexplored.

## Issues of Measurement

Notwithstanding the preliminary research evidence, a validated measurement tool is needed to render processes of PTRG accessible to quantitative investigation. We therefore sought to develop and validate a measurement scale of PTRG. One approach could be to use an existing measure of relationship components and assess relationship growth in these domains, but such scales are often grounded in specific relationship theories and would offer only a limited range of potential relationship growth domains (see Fletcher et al., 2000). We developed our scale to be theoretically integrative, so as to capture relationship qualities that are consistent across major relationship theories (e.g., trust within a relationship is seen as critical in both attachment theory and interdependence theory; Campbell & Stanton, 2019), as well as potential growth-relevant domains identified in qualitative relationship research, relationship resilience research, the perceived growth research, and moral repair research.

A further limitation of existing relationship scales is that they do not capture relationship growth *and* decline. Existing scales often focus on trait/stable relationship qualities (e.g., trust; Rempel et al., 1985) that do not allow for cross-sectional or situational assessments of how experiences like transgressions may diminish or improve these relationship qualities. Furthermore, when they do attempt to measure change, the measurement is often framed in terms of growth, a positivity assumption that may not reflect the true trajectory of a relationship. Indeed, critics have argued that growth is overestimated (i.e., illusory growth), but still a real phenomenon, and researchers must conduct better research into growth to obtain reliable estimates of genuine growth (Infurna & Jayawickreme, 2021). One issue is that commonly used growth scales are unipolar response scales (e.g., Hannon et al., 2010; Park et al., 1996; Tedeschi & Calhoun, 1996) which precludes any reporting on perceived decline and raises demand characteristics. Thus, we constructed our PTRG scale to overcome these critical issues, using a balanced growth/decline, bipolar response scale: −3 (“much less of this”) to 0 (“about the same of this”) to +3 (“much more of this”) as a way to mitigate reporting of illusory growth (see Boals & Schuler, 2018).

## Transforming Relationships Through Co-Reflection

Individuals’ experience of PTRG may depend on whether they engage with each other following the wrongdoing and how they work through the incident together. For example, Waldron and Kelley (2005) examined the post-transgression communication processes that led the victimized relationship partner to perceive relationship weakening, normalizing, or strengthening. Victimized relationship partners who reported greater engagement in discussion with offending relationship partners reported greater relationship strengthening. However, whether discussion leads to relationship transformation likely depends on *how* relationship partners discuss the incident because there are different ways to discuss wrongdoings, and not all of them are constructive (Wenzel et al., 2023).

We argue that *co-reflection*—relationship partners talking through the incident together in a constructive and responsive way (Thai et al., 2023; Wenzel et al., 2023)—is likely to lead to strengthened relationships after transgressions. Other researchers have theorized that relationship partners can create good relationships if they listen to each other and are responsive to each other’s needs (Itzchakov et al., 2022). This responsiveness can become self-perpetuating, transforming the overall quality of relationships. In other words, if relationship partners possess or can cultivate effective ways of responding to each other (e.g., responsiveness; Canevello & Crocker, 2010), then their relationships are more likely to be, or become, higher quality. In this manner, relationship partners who engage in co-reflection after transgressions may become better at meeting each other’s needs and resolving conflicts, which can strengthen the relationship. This is consistent with the key principles of interdependence theory that repeated constructive social interactions can prompt relationship partners to adapt to each other, which can become a stable orientation of constructive responding within the relationship (Rusbult & Van Lange, 2003).

However, aside from relationship partners as interdependent actors (“I and you”) mutually shaping their individually held relationship orientations, it is also possible that relationship partners define themselves as “we” (Cruwys et al., 2023). Through co-reflection, relationship partners may co-construct a new shared understanding of their relationship following a wrongdoing (Woodyatt et al., 2022). Dyadic moral repair research highlights that relationship partners can come to a renewed understanding of the relationship by revalidating violated relationship values (Wenzel et al., 2021). Beyond reaffirming the existing relationship structure, it is possible that transgressions—if dealt with by co-reflection—could lead relationship partners to create new shared values (or reaffirm old forgotten values) because they may learn or realize that the way the relationship was operating led to (or did not prevent) the transgression. By addressing transgressions with co-reflection, relationship partners may realize what really matters in their relationship and/or what they want in their relationship, and this understanding may promote a strengthened relationship (D. L. Kelley et al., 2018).

## The Present Research

Extending on past work, we suggest that interpersonal transgressions may represent an opportunity for the strengthening of relationship qualities, or PTRG. Specifically, we propose that the extent to which relationship partners engage in co-reflection will predict the extent to which they experience relationship growth. Our theorizing about the potential benefits of co-reflection and perceptions of relationship growth was not specific to relationship partners’ role in the transgression. Hence, we recruited both victimized and offending relationship partners in the present research. However, we do not recruit relationship dyads as our main interest in the present research was to demonstrate proof of concept rather than model dyadic processes. We conducted a pilot study and two pre-registered studies to test whether co-reflection may have transformative effects on relationships after transgressions by promoting PTRG. All data, and research materials are available at: https://osf.io/fxt83/?view_only=ac29c4d6b54a414c9a74b1d3c8cd6bc0. We pre-registered and report the design, hypotheses, power analyses, data exclusions, measures and analyses of studies 1 and 2 (study 1: https://osf.io/xjhyr/?view_only=da4e2c8ff09c45ba876be197a70a5870; study 2: https://osf.io/ugvm4/?view_only=e1ee23f2d76b4163a77a473606605da1).

## Pilot Study

Before we could assess relationship growth as a potential outcome of co-reflection following a relational wrongdoing, we first sought to develop a scale measuring PTRG. We initiated item development for this scale by conducting a literature review on quantitative relationship research, qualitative relationship research, relationship resilience research, perceived growth research, and moral repair research. In particular, we sought to identify key relationship qualities that may be affected by relational wrongdoing, and the relationship qualities that may experience growth (and decline) as a consequence of how relationship partners respond to relational wrongdoing. We derived eight relationship qualities from this review: mutual support (Jiang et al., 2022); connection (Cruwys et al., 2023; Emery et al., 2021); open communication (Finkel et al., 2017); new possibilities within relationship (D. L. Kelley et al., 2018; Roepke & Seligman, 2015); relationship flexibility (Fergus & Skerrett, 2015); valuing the relationship (Sasaki et al., 2023); trust (Rempel et al., 1985); and shared values (Wenzel et al., 2021). The Supplemental Material contains an overview for each of these relationship qualities.

Because a multi-item representation of each relationship quality allowed for a factor-analytical test of the scale’s dimensionality, we created 4 items to represent each relationship quality. The reported analyses included all 32 items. However, the observed unidimensionality of our full scale also permitted the creation of a more parsimonious version of our scale. Thus, we also developed and tested a short-form version, selecting one item from each relationship quality whose content appeared to best capture that relationship quality. All analyses with the eight-item version of the PTRG scale reproduced the pattern of results presented in the main text (i.e., across all conducted studies). See Table 1 for all items and both versions of the scales.

**Table 1.** The Post-Transgression Relationship Growth Scale with Reported Growth/Decline Across Items (Pilot Study).

Scale	Experienced growth (%)	Experienced decline (%)
1. Mutual support		
Having compassion for each other	29.7	40.7
Improving each other’s well-being	33.7	35.5
Supporting each other*	44.8	30.2
Making an effort for each other	54.1	27.9
2. Connection		
Having a sense of closeness	29.1	47.7
Feeling connected*	27.9	50.0
Being committed to each other	36.6	33.7
Feeling like one	23.8	51.2
3. Open communications		
Expressing our emotions	51.2	31.4
Listening to each other	49.4	27.9
Communicating openly*	54.7	29.7
Sharing our thoughts	51.7	29.7
4. New possibilities within relationship		
Planning the future together	33.1	33.7
Looking for new things to do together	36.0	34.9
Imagining new possibilities*	32.6	33.7
Setting goals together	33.1	34.9
5. Relationship flexibility		
Coping with problems that arise in our relationship	44.8	26.2
Changing things that need changing in our relationship	54.1	22.1
Finding ways to make things work in our relationship*	57.6	23.3
Trying to make improvements in our relationship	59.3	21.5
6. Valuing the relationship		
Appreciating the value of each other	49.4	27.9
Recognizing the importance of each other	51.2	25.0
Valuing being together*	43.6	27.9
Respecting each other	43.6	28.5
7. Trust		
Trusting each other*	23.8	59.3
Relying on each other	34.9	31.4
Believing in each other	27.9	36.0
Having faith in each other	28.5	44.8
8. Shared values		
Agreeing on what matters in our relationship*	51.2	22.7
Having a shared sense of what binds us together	40.1	29.7
Sharing an understanding of what is important to us	50.6	25.0
Being on the same page about what really matters	48.8	27.9

*Note*. The eight-item version of the scale contains items marked by asterisks.

We conducted a pilot study to test the psychometric qualities and validity of our PTRG scale. A total of 172 residents of the United Kingdom (130 women, 41 men, 1 non-binary) were recruited via Prolific. Participants must have been recently seriously wronged by their relationship partner (i.e., victims) or have seriously wronged their current relationship partner (i.e., offenders) to participate. Our scale presented the following instructions to anchor participants’ responses to the reported wrongdoing and how they engaged with it:

“Have there been any changes in your relationship because of the situation you described and the way your partner and you have responded to it? Please think about the following aspects of relationships and indicate any change in your relationship.—Since this situation and the way we responded to it, there has been. . . .”

To capture the potential growth/decline of relationship qualities, our scale presented responses as −3 (“much less of this”), 0 (“about the same of this”), and +3 (“much more of this”). Table 1 reports the proportion of participants who indicated relationship decline (i.e., scores of −1 to −3) and relationship growth (i.e., scores of +1 to +3) across all 32 items. We conducted principal components analyses on the 32-item PTRG scale in all three studies, and the results were consistent. In general, two or three components emerged from the initial extraction with eigenvalues greater than one. However, the first component explained the majority of variance (69.1%–80.8%). All items loaded strongly onto this component with minimal cross-loadings onto other components. We report these in full detail in the Supplemental Material.

We also examined whether the five-item co-reflection scale of the transgression-related co-rumination scale (Thai et al., 2023) would be positively associated with our newly developed PTRG scale. As anticipated, co-reflection was significantly positively associated with PTRG (*r* = .75), and PTRG was positively associated with relationship satisfaction (*r* = .67; Renshaw et al., 2011), relationship commitment (*r* = .59; Rusbult et al., 1998), hope for a positive future relationship (*r* = .73), and life satisfaction (*r* = .25; Diener et al., 1985; all *p*s < .001). The correlations tended to be sizable but were all <.80, thus posing no threat to discriminant validity (Rönkkö & Cho, 2022). The Supplemental Material includes a detailed description of the method and other results from this pilot study.

The results of the pilot study were promising for the associations between co-reflection and PTRG and the psychometric evaluation of the PTRG scale. However, the cross-sectional design limits confidence in whether co-reflection promotes relationship growth, and whether the subjective PTRG reporting truly represents growth over time. Indeed, perhaps the biggest limitation in the growth literature is the over-reliance on cross-sectional designs (Infurna & Jayawickreme, 2021). For our main studies, we therefore adopted longitudinal research designs to provide evidence for the role of co-reflection in promoting PTRG, and to further validate the PTRG scale.

## Study 1

We conducted Study 1 as a pre-registered longitudinal assessment of whether the co-reflection that occurs in the period after relational wrongdoing can foster PTRG. We examined the prospective effect of co-reflection on PTRG (and vice versa) while controlling for baseline measures. Participants were surveyed over three timepoints: (T1) within 24 hrs of the wrongdoing; (T2) 1 week later; (T3) a further 1 week later. The T1 survey served as initial contact that allowed the recruitment of participants immediately after a transgression and their prospective follow-ups. T1 measures served as baseline assessments of relationship qualities and engagement; because at this point little time would have passed for any continuous co-reflection and any relationship growth to have occurred, these were measured as static relationship characteristics and used as controls. Thus, our pre-registered hypothesis was that T2 co-reflection would be prospectively positively related to T3 PTRG (while controlling for T1 measures).

### Method

#### Participants

We conducted a Monte Carlo simulation power analysis in Mplus v8.4 (Muthén & Muthén, 2017). This determined that we would need 245 participants to detect a standardized coefficient of 0.20 with a power >0.80 for our predicted cross-lagged effect of co-reflection at T2 to PTRG at T3. We pre-registered our decision to recruit 400 participants (i.e., 200 victims and 200 offenders) at T1 to account for expected attrition over the three timepoints.

We launched two surveys (i.e., one advertised as a study for victims, and one advertised for offenders) concurrently on the research platform CloudResearch to obtain an equal quota of victims and offenders at T1. Each survey was only available for 24 hrs to ensure all participants reported on the transgression within a similar timeframe. The surveys were set up to prevent participants from completing both surveys. We screened sign-ups with our pre-registered inclusion criteria that they must have either experienced wrongdoing by their relationship partner (i.e., victims) or wronged their relationship partner (i.e., offenders), within the last 24 hrs. Sign-ups were also screened out if they perceived the severity of the wrongdoing to be less than moderately serious on a 7-point scale from 1 (*not at all serious*) to 7 (*extremely serious*). We excluded sign-ups who reported minor transgressions because this study only focused on a single transgression and relationship partners may not actively address one-off minor transgressions. Sign-ups to the offender study must have indicated that their relationship partner knew about the offense to allow for the possibility of addressing the wrongdoing.

We had the following pre-registered exclusion criteria for our survey. We removed participants’ data if they failed more than one of three embedded attention checks at T1 (*N* = 9), or if any of their responses across the three surveys were incomplete or included wrongdoing descriptions that were not related to a wrongdoing, blank, random text, or described being wronged in the offender study (and vice versa for victims; *N* = 42). The remaining exclusions were due to participants not completing all three timepoints (*N* = 165).^1^ After exclusions, data from 184 participants (108 offenders, 76 victims) were eligible for analysis. Participants were residents of the United States (94 men, 88 women, 1 non-binary, 1 non-specified other; *M*_age_ = 39.2); 71.7% were White, 14.1% Black/African American, 7.6% Hispanic or Latino, 2.7% Asian, 2.7% Multiracial, 0.5% Native American or Alaska Native, and 0.5% non-specified other. Given that the final sample size fell short of the target sample size, we conducted a sensitivity analysis using Monte Carlo simulation in MPlus. It showed that with a sample of *N* = 184, a standardized coefficient of 0.24 for the predicted cross-lag effect of co-reflection at T2 to PTRG at T3 could be detected with a power of 0.80.

#### Design and Procedure

We asked participants at T1 to provide a description of the wrongdoing, to indicate their relationship status (50% long-term relationship, 45.1% married, 3.8% casual dating, 1.1% other), to indicate the duration of their relationship (28.3% more than 10 years, 16.8% 6–10 years, 26.1% 3–5 years, 15.8% 1–2 years, 7.6% 7–12 months, 5.4% 1–6 months), and to categorize the type of wrongdoing (46.7% betrayal of trust, 18.5% verbal fight or argument, 10.3% insult, 9.2% infidelity, 7.1% betrayal of confidence, 4.9% rejection, 2.7% other, 0.5% physical abuse).

After completing T1 and checking eligibility, we contacted participants 1 week later (T2), and again, 1 week later (T3). These latter surveys were identical. In these surveys, participants provided a description of the wrongdoing with a prompt that indicated the date the wrongdoing would have occurred. We then requested that participants rate their level of co-reflection with their relationship partner and other exploratory measures.

### Measures

#### Co-Reflection

We used the five-item co-reflection scale of the *transgression-related co-rumination scale* (Thai et al., 2023). The co-reflection scale contains five items (e.g., “My partner and I tried to work past our negative emotions to resolve the incident”; T1, α = .94; T2, α = .95; T3, α = .95). Items were averaged to obtain scale scores.

#### Post-Transgression Relationship Growth

We report the results of the full 32-item PTRG scale measured at T2 and T3 (see Table 1; T2, α = .99; T3, α = .99), but the 8-item version also reproduced the pattern of results (see Supplemental Material). At T1, we used a static version of the PTRG (α = .99) that used the same items, but with strongly disagree-strongly agree scale labels instead of growth labels (e.g., “strongly agree” rather than “much more of this”). This allowed for a baseline assessment of relationship qualities before any opportunity for growth. Items were averaged to obtain scale scores.

### Results

#### Cross-Lagged Panel Model Analyses

Table 2 presents the means, standard deviations, and bivariate correlations between the main dependent variables. Our primary hypothesis was that co-reflection at T2 would be prospectively positively associated with PTRG at T3 while controlling for the cross-time stabilities within these variables. To test our hypothesis, we used IBM SPSS Amos (Arbuckle, 2019) to conduct conventional cross-lagged panel analyses for the T2 and T3 measures of co-reflection and PTRG. The T1 measures of the adapted PTRG scale (static relationship qualities) and co-reflection served as covariates. This led to a saturated model; hence we do not report model fit statistics.

**Table 2.** Means, Standard Deviations, and Bivariate Zero-Order Correlations for Study 1.

Variable	*M* (*SD*)	2	3	4	5	6
1. Co-reflection T1	4.24 (1.57)	.60***	.73***	.40***	.66***	.39***
2. Static RQ T1	5.28 (1.32)	—	.59***	.39***	.58***	.41***
3. Co-reflection T2	5.15 (1.44)	—	—	.53***	.84***	.55***
4. PTRG T2	4.62 (1.25)	—	—	—	.58***	.83***
5. Co-reflection T3	5.32 (1.47)	—	—	—	—	.70***
6. PTRG T3	4.85 (1.40)					—

*Note.* RQ = relationship qualities; PTRG = post-transgression relationship growth.

*** *p* < .001.

**Figure 1.**
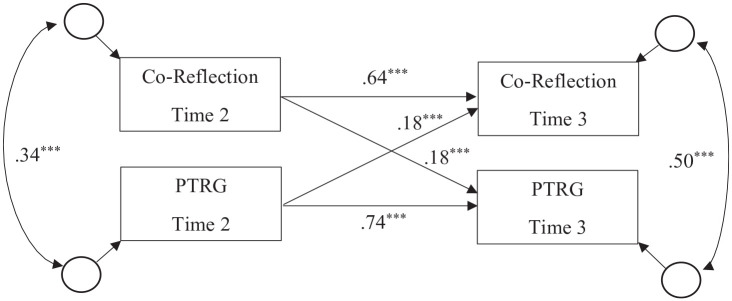
Study 1 standardized coefficients between co-reflection and PTRG. *Note*. The depicted model includes correlations and excludes T1 covariates. PTRG = post-transgression relationship growth. ***p < .001.

We used multigroup analysis to test whether the structural relations between co-reflection and PTRG differed depending on whether the participants were victims or offenders. The chi-square difference test was not statistically significant, χ^2^(24) = 22.9, *p* = .53. This indicated that the fully constrained structural weight model was not significantly different to the baseline unconstrained model, but we note that it is possible the analysis was underpowered to detect differences between the two models. We therefore retained the constrained model as the structural relations were not significantly different between victims and offenders. As predicted, co-reflection at T2 prospectively predicted an increase of PTRG at T3. Examining the reciprocal effect, PTRG at T2 also prospectively predicted an increase of co-reflection at T3 (see Figure 1).^2^ These cross-lagged effects were large effects (Orth et al., 2024).

### Discussion

Study 1 was a pre-registered longitudinal assessment of how PTRG may manifest via the co-reflection that occurs between relationship partners after wrongdoing. We found support for our pre-registered hypothesis that co-reflection at T2 (1 week after the wrongdoing occurred) would prospectively predict an increase of PTRG at T3 (2 weeks after the wrongdoing occurred). This finding speaks to the process of relationship growth after wrongdoing. Namely, co-reflection predicts the trajectory of relationship growth that occurs after relational wrongdoing. This indicates a transformative effect of co-reflection as relationships appear to be changed (for the better) by the degree to which partners discuss and work through the transgression constructively with each other.

A further finding was a reciprocal prospective relationship between co-reflection and PTRG. This suggests that there may be a positive feedback loop between co-reflection and PTRG where co-reflection feeds into PTRG and is fed by PTRG. This may be evidence of how relationships develop the pattern or strategy of how they typically deal with wrongdoing (Zacchilli et al., 2009). Specifically, if relationship partners engage in a constructive manner after wrongdoing (e.g., co-reflection), then this becomes self-reinforcing because it improves the relationship (i.e., PTRG), and an improved relationship makes relationship partners engage with each other in a constructive manner after subsequent wrongdoing, and so on (Canevello & Crocker, 2010).

Overall, the results of study 1 provided support that co-reflection may have transformative effects on relationships. Yet, even though the PTRG items were worded as perceived change (decline or growth) in relationship qualities, it is possible that they just captured a situational snapshot of the current relationship qualities, a static assessment: partners feeling good about their relationship at a point in time may mistakenly perceive this as growth. We needed to ensure that the PTRG scale indeed captures a perception of change and growth in relationship qualities and thereby has surplus value over static assessments. A further limitation of study 1 was the use of a cross-lagged panel model as this can produce biased estimates for the cross-lagged effects especially when wave-specific disturbances/residuals are correlated (Lucas, 2023). These results should be considered preliminary evidence for the theorized relationships given the limitations of this modeling approach. Hence, we conducted latent true change modeling (Steyer et al., 1997) in study 2 to validate the growth component of our scale (i.e., whether it does indeed quantify positive change) and further examine the effects of co-reflection in producing PTRG.

## Study 2

We conducted study 2 with two aims. First, the study served as a pre-registered validation that our PTRG scale taps into perceived growth in the relationship and is not a static trait relationship qualities measure. Toward this purpose, we used latent true change modeling (Steyer et al., 1997), which represents multi-item concepts measured on two occasions as two latent variables: the initial state of the concept at time 1 and the within-person change from time 1 to time 2. The latent initial state draws on time 1 and time 2 measurements of the concept with indicator loadings set to be equal over time, while the latent change draws on time 2 measurements with indicator loadings set to be equal to those of the latent initial state (residuals of repeated indicators are allowed to correlate; for a discussion of the model assumptions, see Geiser et al., 2021). We used this modeling to derive a latent parameter of change from the repeated measurement of the static relationship qualities which we administered at both T1 and T2 in this study. We then examined whether the latent true change score (i.e., representing the growth/decline of the relationship qualities) derived from these measurements predicted perceptions of PTRG measured at T3. That is, we tested whether *measured* (i.e., statistically modeled) change in relationship qualities predicts *perceived* relationship growth. The latent change score modeling allowed measuring change using a relationship qualities scale that tapped into its various dimensions without asking people to report on relationship growth, avoiding the risk that asking about growth might capture illusory perceptions of positive change, a fundamental problem in the perceived growth literature because people are motivated to make positive appraisals of current self/relationships (Infurna & Jayawickreme, 2021). We predicted:

H1: The latent true change score derived from T1 and T2 measurements of the static relationship qualities will be positively associated with PTRG at T3.

In other words, our measure of PTRG at T3 should correspond to actual, statistically modeled improvement in participants’ ratings of the quality of their relationship between T1 and T2. We separated the administration of the PTRG scale by a week (T3) from the relationship growth measured from T1 to T2 to avoid a methodological confounding of measurements.

Second, we sought to conduct a conceptual replication of the findings from Study 1 that co-reflection prospectively predicts increases in PTRG, but now using the latent true change modeling approach. This approach assessed PTRG based on (a) repeated measures of relationship qualities over time and (b) the perceived PTRG scale developed to directly tap subjective perception of growth. Given our use of repeated measures to model growth and only measuring subjective perception of growth once at T3, cross-lagged panel modeling was not suitable in study 2. Instead, we kept to a latent true change modeling approach to derive latent initial state and latent change scores from repeated measurements of both co-reflection and relationship quality. Thus, we could test whether the initial state and change in co-reflection were positively related to modeled (or measured) relationship growth. We predicted:

H2: Reported co-reflection at T1 and T2 (i.e., both the latent initial state score and latent true change score) derived from T1 and T2 measurements of co-reflection will be positively associated with (a) T1 to T2 latent true change in static measures of relationship qualities and (b) with PTRG at T3.

### Method

#### Participants

We aimed to collect the same targeted sample size as in the previous study (i.e., *N* = 245). However, we increased our initial recruitment total from 400 to 450 participants (i.e., 225 victims and 225 offenders) at T1 to account for attrition. We launched two surveys (i.e., one advertised as a study for victims and one advertised for offenders) at the same time on CloudResearch to obtain an equal quota of victims and offenders at T1. Our pre-registered inclusion criteria, screening, and exclusion criteria were the same as study 1 except that we recruited participants on the basis that the wrongdoing had occurred within the last 48 hrs (instead of the 24-hr restriction of study 1) to broaden our potential recruitment pool. However, we only obtained 166 out of a possible 225 responses from offenders within this recruitment time frame. Thus, the initial total sample was 391 participants. We then removed participants’ data who failed more than one of three embedded attention checks (*N* = 3) or if any one of their responses across the three surveys were incomplete or included descriptions of the wrongdoing that were not related to a wrongdoing, blank, random text, or described being wronged in the offender study (and vice versa for victims; *N* = 47). The remaining exclusions were due to participants not completing all three timepoints (*N* = 157).^1^

After exclusions, data from 184 participants (109 offenders, 75 victims) were eligible for analysis. Participants were residents of the United States (104 women, 79 men, 1 non-binary; *M*_age_ = 38.1); 71.2% were White, 11.4% Black or African American, 7.1% Hispanic or Latino, 5.4% Asian, 3.3% Multiracial, and 1.6% Native American or Alaska Native. The final sample size fell short of the target sample size, so we conducted a sensitivity analysis using Monte Carlo simulation in MPlus. It showed that with a sample of *N* = 184, a structural correlation of *r* = .203 could be detected with a power of 0.80; that is, a small to medium effect.

#### Design and Procedure

We asked participants at T1 to provide a description of the wrongdoing, indicate their relationship status (47.8% married, 46.2% long-term relationship, 6.0% casual dating), indicate the duration of their relationship (37.0% more than 10 years, 15.8% 6–10 years, 20.7% 3–5 years, 15.8% 1–2 years, 5.4% 7–12 months, 4.9% 1–6 months, 0.5% less than 1 month), and categorize the type of wrongdoing (44.6% betrayal of trust, 16.8% verbal fight or argument, 15.2% insult, 11.4% infidelity, 4.3% rejection, 3.3% other, 2.7% physical abuse, 1.6% betrayal of confidence). Participants then completed measures of co-reflection and static relationship qualities (i.e., the PTRG scale but adapted to capture static qualities rather than perceived growth in those qualities).

We invited eligible participants back for the T2 survey 1 week later and, again, for the T3 survey another 1 week later. Again, participants provided a description of the wrongdoing. At T2, participants then assessed the level of co-reflection that had occurred between themselves and their relationship partners and completed the measure of static relationship qualities. Finally, at T3, participants completed the perceived PTRG scale with the bi-polar growth/decline scale (i.e., −3 “much less of this” to +3 “much more of this”).

#### Measures

We used 7-point Likert scale (1 = *strongly disagree*, 7 = *strongly agree*) for all items, except the PTRG scale (T3). Participants responded to the PTRG items on a scale presented as −3 (“much less of this”), 0 (“about the same of this”), and +3 (“much more of this”). The same measures from study 1 assessed co-reflection (T1, α = .94; T2, α = .95), static relationship qualities (T1, α = .99; T2, α = .99), and PTRG (T3, α = .99).

### Results

#### Does Change in Relationship Qualities Predict PTRG?

Table 3 presents the means, standard deviations, and bivariate correlations between the main dependent variables. Our first hypothesis test was to assess whether the PTRG scale taps into change/growth in the relationship. To this end, we used *latent true change modeling* (Steyer et al., 1997), a structural equation modeling approach to assess interindividual differences in intraindividual change. We use this approach here to test whether modeled intraindividual change in perceived relationship quality is reflected in PTRG, and whether both modeled and self-reported growth are predicted by co-reflection.

**Table 3.** Means, Standard Deviations, and Bivariate Zero-Order Correlations for Study 2.

Variable	*M* (*SD*)	2	3	4	5
1. Co-reflection T1	4.23 (1.59)	.60***	.67***	.58***	.44***
2. Static RQ T1	5.21 (1.35)	—	.67***	.85***	.56***
3. Co-reflection T2	5.02 (1.46)	—	—	.82***	.63***
4. Static RQ T2	5.23 (1.49)	—	—	—	.69***
5. PTRG T3	4.69 (1.45)	—	—	—	—

*Note.* RQ = relationship qualities; PTRG = post-transgression relationship growth; *SD* = standard deviation.

*** *p* < .001.

**Figure 2.**
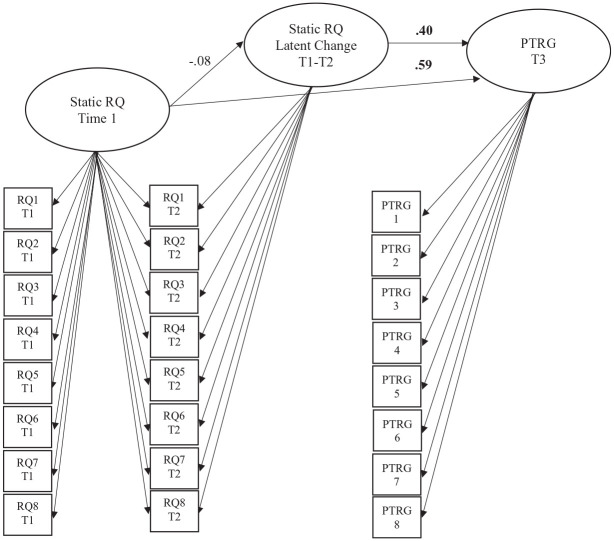
Standardized coefficients for the structural true latent change modeling of static RQ to PTRG. *Note.* Statistically significant relationships at *p* < .001 are bolded. RQ1/PTRG1 = mutual support subscale, etc. (see Table 1). Disturbances, items residuals, and correlations between residuals of matching items across time are omitted from the graph. RQ = relationship qualities; PTRG = post-transgression relationship growth.

Our latent true change model (LTC) model is shown in Figure 2. To test whether modeled change predicts perceived change, the latent change factor of static relationship qualities from T1 to T2 was modeled to predict the PTRG scale (i.e., perceived relationship growth/decline) at T3. We assessed model fit following the recommendations by Hair et al. (2019) and considered multiple fit indices including the chi-square test (χ^2^), an incremental, goodness-of-fit index (comparative fit index [CFI]), and an absolute, badness-of-fit index (standardized root mean residual [SRMR]). The model fit was acceptable, χ^2^(232) = 661.2; CFI = 0.942; SRMR = 0.029. We also used the multigroup analysis function to consider whether victims and offenders differed in the structural relations. The chi-square difference test was non-significant, χ^2^(3) = 5.15, *p* = .16, indicating that the model did not significantly differ for victims and offenders.

We present only the standardized coefficients for the structural LTC model in Figure 2. We depict the measurement model for illustrative purposes. As predicted, the latent change score derived from T1 and T2 measurements of the static relationship qualities was significantly positively associated with PTRG at T3. The latent initial state factor or baseline level of relationship qualities at T1 was also positively associated with PTRG at T3.

#### Does Co-Reflection Predict Change in Relationship Qualities?

We also used latent true change modeling (Steyer et al., 1997) to assess whether the latent initial state factor of co-reflection and the latent true change factor of co-reflection from T1 to T2 predicted the latent change of relationship qualities from T1 to T2.^3^ Thus, we constructed two parallel latent true change models using the T1 and T2 measurements of co-reflection and the static relationship qualities measure, decomposing these into latent initial state factors of co-reflection and static relationship qualities and latent change factors that represent the latent change (growth/decline) in either variable from T1 to T2. The latent initial state factor and latent change factor of co-reflection were then modeled to predict the latent true change factor of static relationship qualities. The model fit was good, χ^2^(290) = 630.7; CFI = 0.952; SRMR = 0.032.

**Figure 3.**
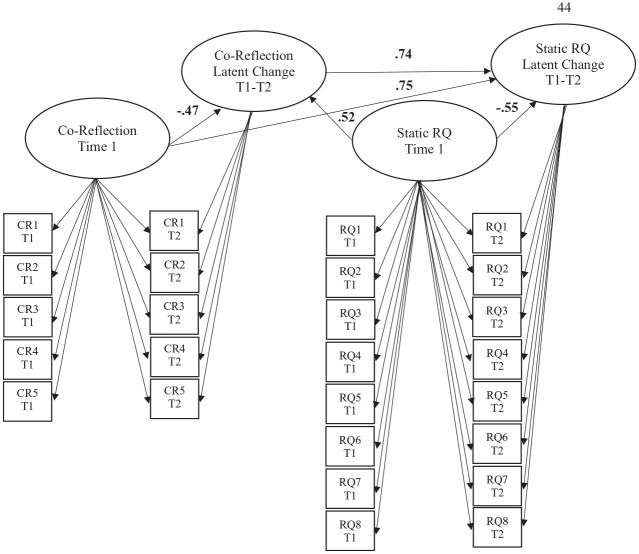
Standardized coefficients for the structural true latent change modeling of co-reflection to the true latent change score of the static relationship qualities scale. *Note.* Statistically significant relationships at *p* < .001 are bolded. RQ1 = mutual support subscale, etc. (see Table 1). Disturbances, items residuals, and correlations between residuals of matching items across time are omitted from the graph. CR = co-reflection; RQ = relationship qualities.

Again, for clarity, we present only the standardized coefficients for the structural LTC model in Figure 3 without the measurement model/factor analysis statistics. In line with predictions (H2a), the latent initial state factor and latent change score of co-reflection were both positively associated with the latent change score in relationship qualities. The latent initial state factor of static relationship qualities was also positively associated with change in co-reflection.

#### Does Co-Reflection Predict PTRG?

**Figure 4.**
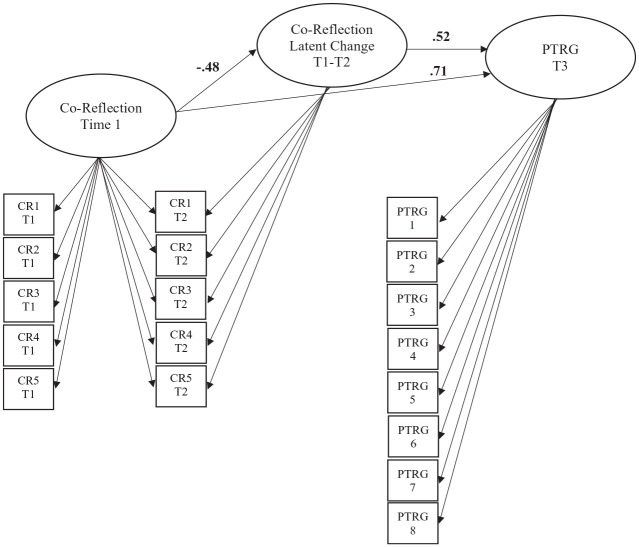
Standardized coefficients for the structural true latent change modeling of co-reflection to PTRG. *Note.* Statistically significant relationships at *p* < .001 are bolded. PTRG1 = mutual support subscale, etc. (see Table 1). Disturbances, items residuals, and correlations between residuals of matching items across time are omitted from the graph. CR = co-reflection; PTRG = post-transgression relationship growth.

Our final use of latent true change modeling was to assess whether the latent initial state factor and latent change factor of co-reflection from T1 to T2 predicted PTRG at T3 (H2b). This latent true change model is shown in Figure 4. The model fit was acceptable, χ^2^(131) = 394.3; CFI = 0.939; SRMR = 0.034. A chi-square difference test that considered whether victims and offenders differed in the structural relations was non-significant, χ^2^(3) = 4.70, *p* = .20. The standardized coefficients for the structural LTC model are presented in Figure 4. As predicted, the latent initial state factor and the latent change score of co-reflection were both significantly positively associated with PTRG at T3.

### Discussion

Study 2 was a pre-registered validation that the PTRG scale taps into growth/decline in relationships after relational wrongdoing. This was demonstrated by deriving a latent change score via the repeated measurement of a static relationship qualities (i.e., a static version of the PTRG items). Findings indicated that this latent change score (i.e., representing the growth/decline of the relationship) predicted the PTRG at the following timepoint. This finding supports the fundamental assumption inherent in the PTRG scale that it assesses growth/decline in the relationship after wrongdoing.

We also extended on the findings of study 1 by further demonstrating the influence of co-reflection in determining relationship growth after wrongdoing. As predicted, both the latent initial state and latent change score derived from repeated measurement of co-reflection were positively associated with *modeled* relationship growth in the form of the latent change derived from the repeated measurement of static relationship qualities, and also prospectively predicted *perceived* relationship growth in form of the PTRG scale at a following timepoint. This finding supports the proposition that the relationships that deal with wrongdoing by engaging in more co-reflection may be the relationships that experience growth.

A further finding was that the baseline level of relationship qualities was positively associated with change in co-reflection. This would suggest that relationships already with stronger relationship qualities (e.g., trust) tend to engage in greater levels of co-reflection over time, possibly being better equipped to overcome initial reluctance (due to threat, or emotional strain) to engage in co-reflection. This finding is consistent with relationship research that conceptualizes relationship qualities such as trust or commitment as representing a confidence in the relationship to be able to successfully address stressful situations such as transgressions and motivating them to do so (Rusbult & Agnew, 2010). It is likely that relationships high in other qualities (e.g., relationship commitment and/or relationship investment) would also be more likely to engage in co-reflection.

## General Discussion

The present research advances the notion that transgressions may be a catalyst for relationship growth if, or when, they are dealt with by relationship partners engaging in the adaptive process of co-reflection (Thai et al., 2023). We found consistent support for the prospective effect of co-reflection on PTRG at subsequent measurements across two three-wave longitudinal studies. This finding supports previous research that demonstrated the benefits of co-reflection for reconciliation (Quinney et al., 2024; Wenzel et al., 2023). However, the present research extends beyond previous understandings of the *reparative* effects of reconciliation on relationships by highlighting that there are potentially *transformative* effects on relationships (Rusbult & Van Lange, 2003); relationships can develop new relationship qualities or be strengthened via the dialogue that relationship partners engage in following transgressions (D. L. Kelley et al., 2018; Waldron & Kelley, 2005).

### Theoretical Contributions and Implications

The current research promotes a transformative lens for understanding moral repair that goes beyond conceptualizing repair as a backward-looking reinstating what was damaged by transgressions. It adds to the growing voice that a reparative lens, with its focus on addressing harm and meeting the needs of victims and offenders, has neglected the growth aspects of repair/healing (Hansen & Umbreit, 2018; Okimoto & Gollwitzer, 2025). Our research highlights the need for research to recognize the varying ways in which relationships might develop or change (for better or worse), including a better understanding of how partners deal with wrongdoing.

The present research also advances the perceived growth literature (Henson et al., 2021) and relationship research (Lee et al., 2018) by highlighting that relationships can experience growth. A key aim of relationship research is to understand why some relationships persist while others decline and dissolve. Given the ubiquity of transgressions in relationships, one important explanation may be how constructively partners deal with transgression situations and whether this affords their relationship to grow from the experience.

As a methodological contribution aligning to this theoretical point, the current research developed and validated the PTRG scale. The PTRG scale is a convenient tool, easily administered in its long or short form, to examine how relationships may experience growth or decline after a transgression. We designed and tested the PTRG scale in ways that overcome shortcomings discussed in the growth literature (Infurna & Jayawickreme, 2021). We constructed our PTRG scale to use a bipolar scale allowing respondents to indicate both relationship decline and growth, which may reduce reports of illusory growth (Boals & Schuler, 2018). For validation, we used longitudinal designs and latent true change modeling (Steyer et al., 1997) in study 2 to assess whether the PTRG scale indeed taps into relationship growth and decline. We repeatedly measured relationship qualities (using a static version of the PTRG to represent the current standing of the relationship on the growth-relevant qualities/domains) and found the latent change score derived from this repeated measurement (an index of relationship change obtained without asking for perceptions of relationship growth which could elicit illusory growth) was positively associated with the PTRG scale. This provides us with confidence that the PTRG scale is a valid measure of relationship growth (the full scale or the short form).

### Limitations and Future Research

The PTRG scale demonstrated unidimensionality despite including items that appear to cover diverse relationship qualities, which may cause concern that the scale brushes over complexity. However, a benefit afforded by its unidimensionality was that we could create an eight-item short form of the scale, which reproduced the pattern of results and provided researchers with a more parsimonious version of the PTRG scale. Second, it is also possible to retain subscales of the full PTRG scale in research contexts where theoretical predictions suggest a more nuanced analysis or where a focus on differences between specific relationship qualities is desired. Third, it is perhaps unsurprising that our PTRG scale is unidimensional because all domains pertain to the relationship and do not refer to other domains that could experience growth, such as personal qualities. Indeed, even other growth-oriented scales with separate personal/relationship growth domains are unidimensional (e.g., Relationship Flourishing Scale; Fowers et al., 2016). As other researchers have noted (Forster et al., 2020), a unidimensional scale does not mean a curtailing of theoretical complexity and scientific progress in understanding the measured phenomenon. Instead, the complexity lies in understanding the conditions under which relationships experience growth (or decline) after transgressions. The PTRG scale may facilitate research on the numerous relationship and transgression-related factors and processes that could condition relationship growth. For example, PTRG may be more (or less) likely to result from different types of transgressions (e.g., infidelity; Perel, 2017).

Another research direction is to recruit relationship dyads. We recruited individual victimized and offending relationship partners, but there are further questions that dyadic data may answer. First, dyadic research may involve observing and coding relationship partners discussing transgressions to understand what aspects of co-reflection are associated with relationship growth. Dyadic research on co-rumination within friendships may suggest that communicating understanding, care, and validation (i.e., responsiveness) could be the working mechanism in co-reflection that leads to relationship growth (Tudder et al., 2024). The recruitment of relationship dyads could also provide independent corroborating evidence that a relationship transgression has indeed occurred and alleviate concerns about data quality in online studies including whether participants genuinely meet inclusion criteria (Guy et al., 2024). Dyadic research could also clarify how much each relationship partner is contributing to co-reflection, and/or what types of intervention (e.g., improving constructive communication patterns through couple therapy; Benson et al., 2012) can improve engagement in co-reflection. Dyadic research may identify that some relationship partners hold divergent views about whether a transgression led to relationship improvement or deterioration. The extent to which relationship partners align in their perceptions of relationship growth may have implications for their shared understanding of the transgression and potentially predict the likelihood of future conflict.

Another limitation of the present research may be the use of self-report. One possible solution may be to use informant reports where people who know the couple (e.g., friends, family) could provide information as to whether they perceive positive or negative changes in the relationship (Blackie et al., 2015). Of course, PTRG is growth that arises from transgressions that occur within the relationship. Thus, there are potential difficulties in using informant reports including whether the couple’s social network has knowledge about the transgression. Further, informant reports would rely on people’s impressions of the relationship/couple and relationship partners may hide relationship difficulties or present as a “happy couple” in public. Informant reports also are biased by what relationship partners choose to share (Frazier et al., 2014). Nevertheless, future investigations incorporating informant reports with these methodological challenges considered could provide additional validation for the PTRG measure.

A future direction is to understand the longer-term trajectory of PTRG over the course of a relationship and recruiting participants before the transgression. Indeed, a limitation of the present research was that we recruited relationship partners after a transgression had occurred and this excluded pre-transgression relationship quality measures. Future longitudinal research should capture baseline assessments of relationship quality (pre-transgression) and then assess whether relationship partners who later experience transgressions and perceive PTRG also report improvement in relationship quality at a later timepoint, after controlling for pre-transgression relationship quality. This would further substantiate that PTRG reflects improvement upon the pre-transgression relationship. Another future research question is whether the occurrence of transgression(s) at different stages in the relationship affects the trajectory of relationship growth differently. For example, early stages of relationships typically experience high levels of growth, but they are also prone to dissolution (Eastwick et al., 2019). Perhaps relationships are prone to slower growth rates or to decline and dissolution if a transgression occurs at an early stage before relationship partners develop key relationship qualities (e.g., trust; Lount et al., 2008) or ways of dealing with incidents (e.g., co-reflection). Alternatively, a transgression at an early stage of a relationship, if effectively dealt with, could be akin to an anabolic steroid affecting significant growth. Relatedly, the development of PTRG over time may not be a continuous linear progression but may have “ups and downs” (Arriaga, 2001). Future research using curvilinear analyses may identify nonlinear development of PTRG over time (Girme, 2020).

We acknowledge that our scale may only be applicable to culturally Western relationships that define relationships by open communication and emotional intimacy. It may also be that direct methods of addressing transgressions (e.g., co-reflection) do not produce PTRG (if transgressions hold such potential across cultures) in collectivistic/interdependent cultures that value indirect communication (Chen et al., 2015; Ge et al., 2022). Future cross-cultural research would need to consider relationship qualities and communicative processes that are relevant within the specific cultural context.

## Conclusion

Transgressions that occur within relationships may be opportunities for relationships to become stronger. The present research demonstrated that co-reflection may have such transformative effects on relationships by leading relationships to develop, improve, or grow. Beyond restoring damaged or broken relationships, relationship partners may work through wrongdoing and believe their relationship is improved from what existed before the wrongdoing; what we have termed PTRG.

## Supplemental Material

sj-docx-1-psp-10.1177_01461672251358619 – Supplemental material for Transformative Moral Repair Following Interpersonal Transgressions: Post-Transgression Relationship Growth Supplemental material, sj-docx-1-psp-10.1177_01461672251358619 for Transformative Moral Repair Following Interpersonal Transgressions: Post-Transgression Relationship Growth by Blake Quinney, Michael Wenzel, Tyler G. Okimoto, Michael Thai and Lydia Woodyatt in Personality and Social Psychology Bulletin

## Notes

1. We conducted the main analyses with the inclusion of the participants who did not complete all timepoints and accounted for their missing data with full information maximum likelihood estimation. However, the pattern of results does not change so we report the analyses without missing data to be consistent with our pre-registration.

2. A concern could be that the positive coefficients do not indicate relationship growth but instead reflect less relationship decline. We created two variables out of the PTRG scale (one to capture relationship growth and another to capture relationship decline) given that the PTRG scale allows for reporting relationship growth, no change, and relationship decline. See the Supplemental Material for details, but co-reflection was positively associated with relationship growth, and negatively associated with relationship decline.

3. A chi-square difference test indicated that the unconstrained and constrained structural weight models were significantly different, χ^2^(5) = 14.8, *p* = .01, but the identified pathways were not critical to our hypothesis, so we retained the model that collapses across both victims and offenders.
