# Supplementary material for: Transformative Moral Repair Following Interpersonal Transgressions: Post-Transgression Relationship Growth
Source: Pers Soc Psychol Bull. 2025 Aug 11;52(9):3017–31. doi: 10.1177/01461672251358619 (PMC13392185; doi:10.1177/01461672251358619)
Supplement: sj-docx-1-psp-10.1177_01461672251358619 – Supplemental material for Transformative Moral Repair Following Interpersonal Transgressions: Post-Transgression Relationship Growth [file sj-docx-1-psp-10.1177_01461672251358619.docx]

**Online Supplementary Material**

***Transformative Moral Repair Following Interpersonal Transgressions: Post-Transgression Relationship Growth***

**Table of Contents**

[**Pilot Study: Item Development** 3](#_Toc181694823)

[**Pilot Study** 7](#_Toc181694824)

[**Method** 8](#_Toc181694825)

[**Results** 11](#_Toc181694826)

[**Discussion** 13](#_Toc181694827)

[**Study 1** 18](#_Toc181694828)

[**Method** 18](#_Toc181694829)

[**Results** 18](#_Toc181694830)

[**Study 2** 24](#_Toc181694831)

[**Results** 24](#_Toc181694832)

[**Supplementary Analyses (8-item PTRG Scale)** 29](#_Toc181694833)

# **Pilot Study: Item Development**

**Mutual Support**

Mutual support includes partner support-related behaviors such as helping each other to achieve personal or shared goals and demonstrating care for each other (Collins & Feeney, 2000; Reis & Clark, 2013). Mutual support can also include holding benevolent attitudes or compassionate goals towards each other, which have been found to be implicated in relationship growth-seeking (Crocker et al., 2017; Jiang et al., 2022). Conversely, perceived decline in partner support after experiencing transgressions may represent a decreased frequency or withdrawal of support (Christensen & Heavy, 1990), or possibly even behaviors that impede each other’s goals and needs such as undermining, sabotaging, criticism, or derogation (Overall et al., 2010).

**Connection**

Relationship partners can view their identity or sense of self as being tied to the other person; they may view themselves as a unit, as one, or as two independent people. Feelings of closeness or connection often correspond to the degree to which relationship partners view themselves as having a shared or fused identity (e.g., Aron et al., 1991; 1992). A sense of shared couple identity has been found to be related to relationship satisfaction (Cruwys et al., 2022); greater perceived clarity of who they are as a couple is associated with commitment (Emery et al., 2021). Thus, growth in this area could represent a stronger inclusion of the other relationship partner in their self-concept or a stronger sense of “we-ness” (Cruwys et al., 2022).

**Open Communication**

Researchers have placed considerable importance on how relationship partners communicate with each other for relationship maintenance and relationship health (Knapp et al., 2020). Effective relationship communication has typically been viewed as involving a perceived openness in how relationship partners respond to each other, such as expressing thoughts/feelings and feeling listened to (Canevello & Crocker, 2010; Itzchakov et al., 2021). Although open communication could involve “positive affect” (e.g., expressing one’s happiness or love), the literature has found that even openly expressing one’s “negative affect” (e.g., expressing anger or disappointment) can be beneficial for relationships because it helps identify issues and bring about change (Overall et al., 2009). Thus, growth in open communications may reflect a perceived improvement in the manner in which the partners are able to talk about relationship problems, which ultimately produces a stronger relationship (for a review, see Finkel et al., 2017).

**New Possibilities within Relationships**

Qualitative evidence suggests that relationship partners can redefine their relationship after serious transgressions, which can involve reconsidering how their relationship should operate moving forward (Kelley et al., 2018). Such redefinition may include relationship partners planning the future and setting goals, but also engaging in novel shared activities, which is linked to increased closeness and romantic relationship excitement (Aron et al., 2000; Coulter et al., 2013). Relationship redefinitions also appear to correspond to the personal growth domain of *new possibilities* in the posttraumatic growth literature where people may perceive new opportunities, pathways, or possibilities to have emerged from dealing with serious major life events (Tedeschi & Calhoun, 1996). We similarly consider the perception of new possibilities within the relationship that may emerge from relationship partners dealing with the transgression.

**Relationship Flexibility**

*Relationship flexibility* is a couple’s ability to adapt to relationship changes and make accommodations within the relationship (Govender, 2022; Fergus & Skerrett, 2015). Relationship flexibility falls under the broad umbrella of relationship resilience in that it captures relationship partners’ ability to rebound or reconfigure after adversity (Lepore & Revenson, 2006). Researchers typically describe relationship flexibility as akin to a “relationship muscle” that experiences growth from successfully dealing with stressors (e.g., Fergus & Skerrett, 2015). Conversely, relationship decline is predicted by relationship inflexibility or an inability to change relationship patterns and dynamics (e.g., continuous engagement in ineffective conflict resolution strategies, see Fincham et al., 2004). We therefore consider perceptions of increased relationship flexibility as another dimension of growth that may derive from relationship partners dealing with transgressions.

**Valuing the Relationship**

Another aspect of our scale is the degree to which relationship partners value being with each other. This includes a relationship partner respecting the other relationship partner and feeling valued/respected themselves, which can be seen as a core aspect of feeling loved in a romantic relationship (Sasaki et al., 2023). The degree to which relationship partners value the relationship is a likely candidate for growth/decline after a transgression because often both the victimized and offending relationship partner feel devalued, disrespected, a lack of integrity, or rejected as a result of wrongdoing (Luchies et al., 2010; McCullough et al., 1997; Shnabel & Nadler, 2008). The quality of dialogue and communication is seen as key to how much a relationship partner is likely to value and respect the other relationship partner and perceive to be valued and respected (Hendrick & Hendrick, 2006).

**Trust**

Relationship partners trusting each other is often regarded by relationship partners as the most important component of a romantic relationship (e.g., Fehr, 1988; Rempel et al., 1985). Trust encapsulates much of what is seen as important in relationships such as feeling safe, dependability, and faith in the partner and the future of the relationship (Campbell & Stanton, 2019). Indeed, a lack of trust tends to be destructive for relationships. For example, relationship partners with low trust in their relationship partner may perceive relationship conflict as more negative and tend to engage in less effective conflict resolution behaviours (Campbell et al., 2010). Growth in trust is likely to arise when relationship partners are seen as engaging in non-selfish accommodations and behaviours benefitting the relationship (Simpson, 2007; Wieselquist et al., 1999). With trust in turn breeding non-selfish accommodating behaviour, this could develop into a learned, response pattern that increases relationship satisfaction and stability over time (Shallcross & Simpson, 2012).

**Shared Values**

Sharing the same values is part of what defines a relationship’s common or shared identity (Wenzel et al., 2008). However, transgressions violate shared values which can disrupt this shared identity (Okimoto & Wenzel, 2008); an unresolved transgression could quite literally decouple the couple identity (Karremans & Van Lange, 2008). Value reaffirmation research typically highlights that working through past transgressions involves the victimized and offending party arriving at a new or renewed understanding of their relationship by coming to a value consensus and revalidating violated values (i.e., a reconsensalization of shared values; Wenzel et al., 2021). Yet, perceived growth in shared values would go beyond repairing or realignment, and instead reflect a perceived reinforcing of these values, or even a recalibrating of values and of what is important and defining of the couple’s shared identity, with implications that things may be done differently in the future.

# **Pilot Study**

We conducted a Pilot study as an initial theory test and construct validation that co-reflection may promote transformative effects on relationships by fostering relationship growth after transgressions. Thus, we predicted that co-reflection would be positively associated with post-transgression relationship growth. Our second aim was to develop the post-transgression relationship growth scale (PTRG) and test its psychometric qualities and validity. Therefore, we predicted that the PTRG scale would be positively associated with other relationship health/quality indices (e.g., relationship satisfaction), and personal life quality (e.g., life satisfaction).

We were also interested in whether PTRG may predict relationship health/quality indices, over and above reparative-based relationship repair outcomes (i.e., victims’ power/status restoration, and offenders’ moral identity restoration). A reparative moral repair lens would specify that relationship repair is achieved via a transactional process of victim and offender addressing each other’s needs and threats (e.g., Shnabel & Nadler, 2008). We aimed to demonstrate that the relationship transformative outcome of PTRG has predictive value above and beyond these reparative-based repair outcomes for relationship health/quality indices (e.g., relationship commitment), future-oriented positive relationship expectations (e.g., hope for a positive future relationship), and victims’ forgiveness/offenders’ self-forgiveness.

## **Method**

**Participants.** We conducted our study on the research platform Prolific. Our inclusion criteria were that participants must have been seriously wronged by their relationship partner (i.e., ‘victims’), or have seriously wronged their current relationship partner (i.e., ‘offenders’). The reported wrongdoing must have occurred at least one week ago, to allow time for relationship partners to address the wrongdoing. However, to ensure recency, the wrongdoing must have occurred no longer than three months ago, so that relationship partners may recall how it was dealt with and the consequences for their relationship. We excluded data from participants if the wrongdoing caused the relationship to dissolve (28 observations excluded), or if a participant indicated the wrongdoing was less than moderately serious on a 7-point Likert-scale (1 = not at all serious, 4 = moderately serious, 7 = extremely serious; 30 observations excluded). We excluded data from offenders if they indicated that their relationship partner did not know about the offense because this would preclude dyadic engagement with the wrongdoing (5 observations excluded). Finally, our survey also contained three embedded attention checks, but no participant failed more than one attention check; we thus retained data from 172 participants (120 victims, 52 offenders). Participants were residents of the United Kingdom (130 female, 41 male, 1 non-binary; M_age_ = 34.7); 83.7% were White/White British, 7.6% Asian/Asian British, 4.1% multiracial, 3.5% Black/African/Caribbean British, and 1.2% Moroccan.

**Design and Procedure.** Participants were first asked to provide a description of the wrongdoing, indicate their relationship status (59.3% long-term relationship, 34.3% married, 5.8% casual dating, 0.6% other), indicate the duration of their relationship (26.7% more than 10 years, 25.6% 6-10 years, 24.4% 3-5 years, 15.7% 1-2 years, 2.9% 7-12 months, 4.7% 1-6 months), and categorize the type of wrongdoing (42.4% betrayal of trust, 27.9% infidelity, 12.8% verbal fight or argument, 5.8% insult, 2.9% physical abuse, 2.9% rejection, 3.5% other, 1.7% betrayal of confidence). Participants then completed the measures in the order described below (with item-order randomized within scales), except the presentation of the post-transgression relationship growth and transactional based restorative outcomes (i.e., victim’s restored status/power, and offender’s restored moral identity) were counterbalanced to avoid order effects, given these were tested as competing predictors.

**Measures.** All items were measured on a 7-point Likert scale (1 = strongly disagree, 2 = disagree, 3 = somewhat disagree, 4 = neither agree nor disagree, 5 = somewhat agree, 6 = agree, 7 = strongly agree), unless otherwise indicated. Items were averaged on all measures to create a single score after reverse-coding relevant items. Only participants who indicated that they were wronged by their relationship partner (i.e., ‘victims’) completed the forgiveness scale and the status/power restoration scale, whereas only participants who indicated that they wronged their relationship partner (i.e., ‘offenders’) completed the self-forgiveness scale and the moral identity restoration scale.

***Transgression-Related Co-Rumination.*** The fifteen-item scale by Thai et al. (2023a) was used to capture participants’ assessment of how they and their relationship partner had engaged with the relational transgression. The scale contains 5 items assessing co-reflection (e.g., “*My partner and I made an effort to see the incident in a different light based on what the other person had to say*”; α = .92), co-brooding (“*My partner and I seemed to get stuck in circles talking about the incident*”; α = .87), and co-avoidance (“*My partner and I tried to talk about anything other than the incident*”; α = .85). The three-factor structure was supported by a principal component analysis that yielded three components that explained 27.9%, 21.4%, and 20.9% of the variance, following a Varimax rotation.

***Relationship Satisfaction.*** We used the generic format of the Relationship Assessment Scale to assess relationship satisfaction (Renshaw et al., 2011). We further adapted the referent on each item to ‘your partner’. Each item on this seven-item scale (e.g., “*In general, how satisfied are you with your relationship with your partner?*”) has item-specific scale anchors (e.g., 1 = not satisfied; 5 = very satisfied; α = .94).

***Relationship Commitment.*** We used the commitment level items from Rusbult et al. (1998) to capture participants’ commitment to their relationship. This scale contains seven items (e.g., “*I am committed to maintaining my relationship with this person*”; α = .93).

***Hope for a Positive Future Relationship*.** Three items assessed the extent to which participants believed their relationship would be positive moving forward: “*How hopeful are you of having a good relationship with this person in the future?*”; “*How hopeful are you that the relationship will grow further in the future?*”; “*How hopeful are you that the relationship will be resilient to challenges in the future?*” (1 = not at all; 7 = very much so; α = .97).

***Life Satisfaction Scale.*** We assessed participants’ life satisfaction with the Satisfaction with Life Scale (Diener et al., 1985). The Satisfaction with Life Scale has seven items (e.g., “*In most ways my life is close to my ideal*”; α = .92).

***Status/Power Restoration* (Victims only).** Three items assessed the extent to which victims felt their need for status/power restoration had been met by the offender: “*I feel respected by the other person*”; “*The other person makes me feel esteemed*”; “*The other person shows humility towards me*” (α = .85).

***Moral Identity Restoration* (Offenders only).** Three items assessed the extent to which offenders felt their need for morality and social acceptance had been met by the victim: “*I feel accepted by the other person*”; “*I feel forgiven by the other person*”; “*The other person makes me feel like a good/moral person*” (α = .88).

***Forgiveness* (Victims only)*.*** We used the 18-item Transgression-Related Interpersonal Motivation scale (TRIM-18; McCullough et al., 1998) to assess victims’ forgiveness. Originally, the TRIM-18 was divided into three subscales (i.e., benevolence, revenge, and avoidance). However, other research has found that forgiveness can be represented as a single dimension (Forster et al., 2020). Indeed, the internal consistency for all 18 items was high in this study (α = .86).

***Self-Forgiveness* (Offenders only).** We used the Differentiated Process Scale of Self-Forgiveness (Woodyatt & Wenzel, 2013) which measures offenders’ *genuine self-forgiveness* (7 items, e.g., “*Since committing the offense I have tried to change*”; α = .92), *pseudo self-forgiveness* (6 items, e.g., “*I wasn’t the only one to blame for what happened*”; α = .86), and *self-punitiveness* (7 items, e.g., “*I want to punish myself for what I have done*”; α = .94). We reworded items with the referent ‘the other person’ to ‘my partner’.

## **Results**

***Exploratory Factor Analysis of the Post-Transgression Relationship Growth Scale***

A principal components analysis was conducted to explore the component structure of the 32-item post-transgression relationship growth scale. Supplementary Table 1 presents the results. Bartlett’s Test of Sphericity indicated that the correlation matrix was not random, χ^2^(496) = 6981.87, p <.001, and the Kaiser–Meyer–Olkin’s (KMO) was above the minimum of .50 (KMO = .97). Three components emerged from the initial extraction with eigenvalues greater than one, and as observed on the scree plot. However, the first component explained 69.1% of the variance with the latter explaining 4.5% and 3.2%, respectively. All items strongly loaded onto the first factor (standardized loadings >.70), and only several items cross loaded onto the other two factors (< .45). The 32 items showed very high internal consistency (α = .99), and thus a composite scale score was created by averaging all items.

***Convergent*** ***and Predictive Validity***

Supplementary Table 2 presents the means, standard deviations, and zero-order bivariate correlations between all composite scores. First, we expected positive associations between our post-transgression relationship growth scale (PTRG) and other relationship quality indices. Indeed, PTRG was positively associated with relationship satisfaction, relationship commitment, and life satisfaction. This provided initial evidence of convergent validity for our PTRG scale.

We were also interested in whether co-reflection may predict PTRG. As seen in Supplementary Table 2, co-reflection was positively associated with PTRG. We also examined the other aspects of post-transgression co-rumination and found that co-brooding was negatively associated with PTRG, but co-avoidance was not significantly associated with PTRG.

We also conducted simultaneous multiple regression analyses to determine whether PTRG would predict relationship measures (e.g., relationship commitment), and future-oriented positive relationship expectations (e.g., hope for a positive future relationship), over and above reparative-based restorative processes (i.e., victims’ status/restoration, and offenders’ moral identity restoration). We split the datafile into victims and offenders for these analyses. Supplementary Table 3 contains the standardized coefficients. As expected, PTRG was a significant positive predictor of hope for a future positive relationship, relationship satisfaction, and relationship commitment, for both victims and offenders. Moreover, the unique contributions of status/power restoration (victims), and moral identity restoration (offenders) towards these outcomes were non-significant. For victims, both PTRG and status/power were significant positive predictors of forgiveness. For offenders, PTRG was a significant positive predictor of genuine self-forgiveness, and a negative predictor of pseudo self-forgiveness, but PTRG did not significantly predict self-punitiveness. Moral identity restoration had significant unique contributions, but it was a significant *negative* predictor of genuine self-forgiveness, a *positive* predictor of pseudo self-forgiveness, and a *negative* predictor of self-punitiveness.

## **Discussion**

The pilot study provided initial support for the reliability and validity of our PTRG scale. PTRG had strong positive associations with other relationship quality measures (i.e., relationship satisfaction, and relationship commitment), and a moderate positive association with life satisfaction. However, we also note that these correlations were all < .80, therefore posing no threat to discriminant validity (Rönkkö & Cho, 2022). Further, co-reflection was positively associated with PTRG providing initial support for our theorising that relationship growth after wrongdoing may be a function of *how* relationship partners address the wrongdoing.

Another finding of this study was that PTRG was positively associated with relationship outcomes (e.g., relationship satisfaction), hope for a positive future relationship, and victims’ forgiveness/offenders’ self-forgiveness, over and above reparative-based outcomes (i.e., status/power restoration for victims, and moral identity restoration for offenders). Research drawing on reparative models of reconciliation typically emphasize reconciliation as a process of victims and offenders reciprocally meeting each other’s psychological needs to address what was damaged by the transgression (Shnabel & Nadler, 2008, 2015). Yet, our findings may suggest that relationship partners become (re)committed to their relationship by perceiving the relationship to have grown after wrongdoing, rather than outcomes that have a backward-looking focus on repairing what was damaged by the transgression.

The results of the pilot study were promising for the associations between co-reflection and PTRG and the psychometric evaluation of our PTRG scale. However, the cross-sectional design limits the confidence in whether co-reflection causes relationships to grow or decline, as well as the growth aspect of our scale. Indeed, perhaps the biggest limitation in the growth literature is the over-reliance on cross-sectional designs (Infurna & Jayawickreme, 2019; Jayawickreme & Blackie, 2014). To address these limitations, we adopted longitudinal research designs to provide further evidence for the co-reflection to PTRG link, and to further substantiate our PTRG scale.

**Supplementary Table 1.** The Post-Transgression Relationship Growth Scale and PCA Component Loadings in Pilot Study

| Scale | Loadings | Scale | Loadings |
| --- | --- | --- | --- |
| **Mutual Support** |  | **Relationship Flexibility** |  |
| Having compassion for each other. | 0.76 | Coping with problems that arise in our relationship. | 0.79 |
| Improving each other’s well-being. | 0.79 | Changing things that need changing in our relationship. | 0.80 |
| Supporting each other. | 0.81 | Finding ways to make things work in our relationship. | 0.82 |
| Making an effort for each other. | 0.83 | Trying to make improvements in our relationship. | 0.83 |
|  |  |  |  |
| **Connection** |  | **Valuing the Relationship** |  |
| Having a sense of closeness. | 0.81 | Appreciating the value of each other. | 0.89 |
| Feeling connected. | 0.82 | Recognizing the importance of each other. | 0.90 |
| Being committed to each other. | 0.83 | Valuing being together. | 0.87 |
| Feeling like one. | 0.80 | Respecting each other. | 0.86 |
|  |  |  |  |
| **Open communications** |  | **Trust** |  |
| Expressing our emotions. | 0.85 | Trusting each other. | 0.72 |
| Listening to each other. | 0.85 | Relying on each other. | 0.82 |
| Communicating openly. | 0.88 | Believing in each other. | 0.88 |
| Sharing our thoughts. | 0.84 | Having faith in each other. | 0.81 |
|  |  |  |  |
| **New Possibilities within Relationship** |  | **Shared Values** |  |
| Planning the future together. | 0.83 | Agreeing on what matters in our relationship. | 0.88 |
| Looking for new things to do together. | 0.81 | Having a shared sense of what binds us together. | 0.87 |
| Imagining new possibilities. | 0.80 | Sharing an understanding of what is important to us. | 0.86 |
| Setting goals together. | 0.81 | Being on the same page about what really matters. | 0.85 |

**Supplementary Table 2.** Means, Standard Deviations, and Bivariate Zero-Order Correlations for all Variables in Pilot Study

| Variable | *M* (*SD*) | 2 | 3 | 4 | 5 | 6 | 7 | 8 | 9 | 10 | 11 | 12 | 13 | 14 |
| --- | --- | --- | --- | --- | --- | --- | --- | --- | --- | --- | --- | --- | --- | --- |
| 1. PTRG | 4.05 (1.42) | .75^***^ | -.47^***^ | -.15 | .67^***^ | .59^***^ | .25^***^ | .73^***^ | .75^***^ | .70^***^ | .74^***^ | .35^*^ | -.20 | -.16 |
| 2. Co-Reflection | 4.44 (1.42) | - | -.58^***^ | -.21^**^ | .60^***^ | .53^***^ | .26^***^ | .66^***^ | .70^***^ | .62^***^ | .78^***^ | .36^**^ | -.26 | -.10 |
| 3. Co-Brooding | 4.55 (1.35) | - | - | .09 | -.38^***^ | -.25^***^ | -.20^**^ | -.34^***^ | -.55^***^ | -.49^***^ | -.41^**^ | -.14 | .27 | .07 |
| 4. Co-Avoidance | 3.10 (1.27) | - | - | - | -.25^***^ | -.16^*^ | -.14 | .22^**^ | -.14 | -.17 | .09 | -.24 | .35^*^ | -.01 |
| 5. Relationship Satisfaction | 4.94 (1.47) | - | - | - | - | .77^***^ | .40^***^ | .78^***^ | .58^***^ | .67^***^ | .29^*^ | .43^**^ | -.43^**^ | -.03 |
| 6. Relationship Commitment | 5.56 (1.49) | - | - | - | - | - | .21^**^ | .78^***^ | .49^***^ | .71^***^ | .32^*^ | .42^**^ | -.35^*^ | -.07 |
| 7. Life Satisfaction | 4.29 (1.44) | - | - | - | - | - | - | .30^***^ | .33^***^ | .23^*^ | .16 | .11 | -.17 | -.07 |
| 8. Hope for a Positive Future Rel. | 5.12 (1.83) | - | - | - | - | - | - | - | .63^***^ | .74^***^ | .51^***^ | .33^*^ | -.31^*^ | -.14 |
| 9. Status/Power Restoration (V) | 3.30 (1.47) | - | - | - | - | - | - | - | - | .62^***^ | - | - | - | - |
| 10. Forgiveness (V) | 5.46 (1.16) | - | - | - | - | - | - | - | - | - | - | - | - | - |
| 11. Moral Identity Restoration (O) | 4.10 (1.50) | - | - | - | - | - | - | - | - | - | - | .02 | .06 | -.35^*^ |
| 12. Genuine Self-Forgiveness (O) | 5.04 (1.39) | - | - | - | - | - | - | - | - | - | - | - | -.66^***^ | .60^***^ |
| 13. Pseudo Self-Forgiveness (O) | 2.70 (1.33) | - | - | - | - | - | - | - | - | - | - | - | - | -.44^***^ |
| 14. Self-Punitiveness (O) | 3.92 (1.63) | - | - | - | - | - | - | - | - | - | - | - | - | - |

*Note.* *p < .05, **p < .01, ***p < .001. PTRG = post-transgression relationship growth, Rel = relationship, V = victim only, O = offender only.

**Supplementary Table 3.** Standardized Coefficients for the Simultaneous Multiple Regressions Conducted in Pilot Study

|  | Hope for a  Future Positive Rel. | |  | Relationship Satisfaction | |  | Relationship Commitment | |  | Forgiveness | |  | Genuine  Self-Forgiveness | |  | Pseudo  Self-Forgiveness | |  | Self-Punitiveness | |
| --- | --- | --- | --- | --- | --- | --- | --- | --- | --- | --- | --- | --- | --- | --- | --- | --- | --- | --- | --- | --- |
|  | β | *p* |  | β | *p* |  | β | *p* |  | β | *p* |  | β | *p* |  | β | *p* |  | β | *p* |
| Offenders |  |  |  |  |  |  |  |  |  |  |  |  |  |  |  |  |  |  |  |  |
| PTRG | **.54** | **.002** |  | **.76** | **< .001** |  | **.72** | **< .001** |  | - | *-* |  | **.74** | **< .001** |  | **-.53** | **.01** |  | .23 | .26 |
| Moral Identity  Restoration | .11 | .50 |  | -.27 | .12 |  | -.22 | .22 |  | - | *-* |  | **-.53** | **.006** |  | **.45** | **.03** |  | **-.51** | **.01** |
|  |  |  |  |  |  |  |  |  |  |  |  |  |  |  |  |  |  |  |  |  |
| Victims |  |  |  |  |  |  |  |  |  |  |  |  |  |  |  |  |  |  |  |  |
| PTRG | **.63** | **< .001** |  | **.56** | **< .001** |  | **.46** | **< .001** |  | **.53** | **< .001** |  | - | - |  | - | - |  | - | - |
| Status/Power  Restoration | .22 | .07 |  | .16 | .12 |  | .14 | .21 |  | **.23** | **.02** |  | - | - |  | - | - |  | - | - |

*Notes.* Statistically significant relationships are bolded. PTRG = post-transgression relationship growth scale; rel. = relationship.

# **Study 1**

## **Method**

***Measures***

**Relationship Growth.** We included four items as a direct measure of relationship growth to assess the construct validity of our PTRG scale: “*Due to how my partner and I have responded to the wrongdoing, my relationship with my partner has grown*”; “*Due to how my partner and I have responded to the wrongdoing, my relationship with my partner has improved*”; “*Due to how my partner and I have responded to the wrongdoing, my relationship with my partner has deteriorated*” (reverse-coded); “*Due to how my partner and I have responded to the wrongdoing, my relationship with my partner has declined*” (reverse-coded; T2, α = .92; T3, α = .94).

## **Results**

***Factor Analysis of the Post-Transgression Relationship Growth Scale***

We conducted principal components analyses (PCA) on the 32-item static PTRG scale and PTRG scale. The results mirror the PCA conducted in Study 1. First, across all three measurements, Bartlett’s Test of Sphericity indicated that the correlation matrix was not random, and the Kaiser–Meyer–Olkin’s (KMO) was above the minimum of .50. Three components emerged from the initial extraction with eigenvalues greater than one (two for T3). Again, like Study 1, the first component explained a large amount of the variance (T1: 73.0%; T2: 75.1%; T3: 80.8%). All items loaded strongly onto the first factor. See Supplementary Table 4.

**Descriptive Statistics.** Supplementary Table 5 presents the relationships between post-transgression relationship growth (PTRG) to the other self-forgiveness scales (pseudo self-forgiveness, and self-punitiveness). Only pseudo self-forgiveness at T1 was significantly negatively associated with PTRG at T2.

**T1 Covariates.** We note that the main cross-lagged panel analysis reported in the main manuscript does not report on the T1 covariates. We note here these relationships. For T1 static relationship qualities as the covariate: T1 static relationship qualities to T2 co-reflection (β = 0.24, *p* < .001), T1 static relationship qualities to T2 PTRG (β = 0.23, *p* = .005), T1 static relationship qualities to T3 co-reflection (β = 0.09, *p* = .060), T1 static relationship qualities to T3 PTRG (β = 0.06, *p* = .221). For co-reflection at T1 as the covariate: T1 co-reflection to T2 PTRG (β = 0.26, *p* = .002), T1 co-reflection to T2 co-reflection (β = 0.59, *p* < .001), T1 co-reflection to T3 PTRG (β = -0.07, *p* = .271), T1 co-reflection to T3 co-reflection (β = 0.08, *p* = .197).

**Model Comparison Between Cross-Lagged Panel Model with and without Covariates.** We also conducted a cross-lagged panel model without covariates to compare how the cross-lagged relationships may differ depending on whether covariates are included in the analysis. Supplementary Table 6 presents the results. Both models demonstrated the same pattern of results.

**Scale Validation Cross-Lagged Panel Model Analyses.** We also sought to further validate our PTRG scale by substituting our PTRG scale with the directly worded relationship growth scale. The model was saturated and so we do not report model fit. As depicted in Supplementary Figure 1, the observed cross lagged effects between co-reflection and the relationship growth scale are consistent with the observed cross lagged effects between co-reflection and our PTRG scale. Namely, co-reflection at T2 prospectively predicts an increase in relationship growth at T3. Moreover, relationship growth at T2 prospectively predicts an increase of co-reflection at T3.

.14^*^

.61^***^

21^***^

.71^***^

**Supplementary Figure 1.** Standardized coefficients between co-reflection and relationship growth.

Note. The model excludes within-wave correlations of residual variances. Statistically significant cross-lagged relationships are bolded. ^*^ p < .05, ^***^ p < .001

**Exploratory Cross-Lagged Panel Model Analyses.** We constructed four cross-lagged panel models to examine the prospective relationships between PTRG and victims’ forgiveness (see Supplementary Figure 2), and between PTRG and offenders’ self-forgiveness (including a separate model for genuine self-forgiveness, pseudo self-forgiveness, and self-punitiveness). We used the adapted PTRG (static relationship qualities) and forgiveness/self-forgiveness measurements at T1 to serve as covariates, hence the models were saturated. However, there were no significant prospective effects of PTRG to forgiveness/self-forgiveness, nor significant prospective effects of forgiveness/self-forgiveness to PTRG (except T2 pseudo self-forgiveness was significantly negatively associated with PTRG at T3; see Supplementary Figure 3).

.07

.74^***^

.12

.81^***^

**Supplementary Figure 2.** Standardized coefficients between post-transgression relationship growth and forgiveness.

Note. The model excludes within-wave correlations of residual variances. ^***^ p < .001

(a)

.08

.77^***^

.07

.63^***^

(b)

-.01

.83^***^

-.18^*^

.58^***^

(c)

.07

.80^***^

-.05

.67^***^

**Supplementary Figure 3.** Standardized coefficients between post-transgression relationship growth and genuine self-forgiveness (a), pseudo self-forgiveness (b), self-punitiveness (c)

**Post-Transgression Relationship Growth or Less Relationship Decline?**

One alternative reading of our results could be that the scores do not necessarily reflect post-transgression relationship growth but instead may reflect a decrease in post-transgression relationship decline (i.e., the positive coefficient may reflect variance between scores that correspond to relationship decline). We tested this possibility by creating two variables out of the PTRG scale given that the Bi-polar scale captured reported relationship growth, no change, and relationship decline. We created one variable to reflect reported post-transgression relationship growth (PTRG), that is coded 0 for any score at or *below* the midpoint of the scale (the midpoint corresponds to no reported change – “about the same of this”), and 1-3 for reported growth (e.g., 3 = “much more of this”). The other variable captured reported post-transgression relationship decline (PTRD), as we coded 0 for any score at or *above* the midpoint of the scale and 1-3 for reported decline (e.g., 3 = “much less of this”). We re-ran the analysis with both PTRG and PTRD entered simultaneously. Consistent with results in the main manuscript, there was a significant positive path coefficient from T2 co-reflection to T3 PTRG (β = 0.22, *p* < .001). There was a significant *negative* path coefficient from T2 co-reflection to T3 post-transgression relationship decline (β = -0.23, *p* < .001). There was also a significant negative path coefficient from T2 PTRD to T3 co-reflection (β = -0.19, *p* < .001). Supplementary Figure 4 presents the results.

.62^***^

.02 / -.19^***^

.22^***^ / -.23^***^

.72^***^/.66^***^/

**Supplementary Figure 4.** Standardized coefficients between co-reflection and post-transgression relationship growth (PTRG), and co-reflection and post-transgression relationship decline (PTRD).

Notes. Statistically significant cross-lagged relationships are bolded. ^*^ p < .05, ^***^ p < .001. The model excludes the T1 covariates and within-wave correlations.

**Supplementary Table 4.** The Post-Transgression Relationship Growth Scale and PCA Component Loadings Study 1

| Scale | Loadings (T1, T2, T3) | Scale | Loadings (T1, T2, T3) |
| --- | --- | --- | --- |
| **Mutual Support** |  | **Relationship Flexibility** |  |
| Having compassion for each other. | 0.84, 0.89, 0.89 | Coping with problems that arise in our relationship. | 0.82, 0.82, 0.85 |
| Improving each other’s well-being. | 0.86, 0.88, 0.88 | Changing things that need changing in our relationship. | 0.86, 0.82, 0.86 |
| Supporting each other. | 0.88, 0.90, 0.92 | Finding ways to make things work in our relationship. | 0.84, 0.89, 0.91 |
| Making an effort for each other. | 0.89, 0.88, 0.90 | Trying to make improvements in our relationship. | 0.89, 0.89, 0.90 |
|  |  |  |  |
| **Connection** |  | **Valuing the Relationship** |  |
| Having a sense of closeness. | 0.87, 0.89, 0.92 | Appreciating the value of each other. | 0.91, 0.91, 0.92 |
| Feeling connected. | 0.87, 0.91, 0.92 | Recognizing the importance of each other. | 0.91, 0.88, 0.93 |
| Being committed to each other. | 0.81, 0.88, 0.91 | Valuing being together. | 0.91, 0.89, 0.93 |
| Feeling like one. | 0.85, 0.86, 0.91 | Respecting each other. | 0.90, 0.88, 0.94 |
|  |  |  |  |
| **Open communications** |  | **Trust** |  |
| Expressing our emotions. | 0.77, 0.80, 0.84 | Trusting each other. | 0.84, 0.86, 0.87 |
| Listening to each other. | 0.90, 0.85, 0.89 | Relying on each other. | 0.86, 0.88, 0.91 |
| Communicating openly. | 0.83, 0.83, 0.88 | Believing in each other. | 0.89, 0.89, 0.92 |
| Sharing our thoughts. | 0.83, 0.82, 0.87 | Having faith in each other. | 0.89, 0.87, 0.92 |
|  |  |  |  |
| **New Possibilities within Relationship** |  | **Shared Values** |  |
| Planning the future together. | 0.81, 0.81, 0.86 | Agreeing on what matters in our relationship. | 0.84, 0.87, 0.89 |
| Looking for new things to do together. | 0.79, 0.81, 0.89 | Having a shared sense of what binds us together. | 0.86, 0.89, 0.92 |
| Imagining new possibilities. | 0.83, 0.86, 0.91 | Sharing an understanding of what is important to us. | 0.88, 0.88, 0.93 |
| Setting goals together. | 0.79, 0.85, 0.89 | Being on the same page about what really matters. | 0.84, 0.89, 0.89 |

*Notes.* The T1 measurement is the static worded scale version.

**Supplementary Table 5.** Means, Standard Deviations, and Bivariate Zero-Order Correlations for Offenders Study 1

|  |  |  | Static RQ PTRG | | | Pseudo SF | | | Self-Punitiveness | | |
| --- | --- | --- | --- | --- | --- | --- | --- | --- | --- | --- | --- |
| Variable |  | *M* (*SD*) | T1 | T2 | T3 | T1 | T2 | T3 | T1 | T2 | T3 |
|  |  |  |  |  |  |  |  |  |  |  |  |
| Static RQ T1 |  | 5.38 (1.18) | - |  |  |  |  |  |  |  |  |
|  |  |  |  |  |  |  |  |  |  |  |  |
| PTRG T2 |  | 4.67 (1.20) | .46^***^ | - |  |  |  |  |  |  |  |
| PTRG T3 |  | 4.94 (1.31) | .40^***^ | .82^***^ | - |  |  |  |  |  |  |
|  |  |  |  |  |  |  |  |  |  |  |  |
| Pseudo SF T1 |  | 2.86 (1.39) | -.50^***^ | -.23^**^ | -.17 | - |  |  |  |  |  |
| Pseudo SF T2 |  | 2.89 (1.32) | -.41^***^ | -.06 | -.13 | .71^***^ | - |  |  |  |  |
| Pseudo SF T3 |  | 2.71 (1.26) | -.38^***^ | -.11 | -.17 | .77^***^ | .83^***^ | - |  |  |  |
|  |  |  |  |  |  |  |  |  |  |  |  |
| Self-Punitiveness T1 | | 3.31 (1.51) | -.08 | -.01 | .06 | -.07 | -.01 | -.04 | - |  |  |
| Self-Punitiveness T2 |  | 3.24 1.57) | -.18 | .01 | .03 | -.06 | .05 | .01 | .75^***^ | - |  |
| Self-Punitiveness T3 |  | 3.09 (1.58) | -.06 | .09 | .08 | -.14 | .01 | .01 | .66^***^ | .79^***^ | - |

*Notes.* RQ = relationship qualities, PTRG = post-transgression relationship growth, SF = self-forgiveness

**Supplementary Table 6.** Model Comparison Between Cross-Lagged Models with and without Covariates

| *Model with Covariates* | | |  | b | *SE* | *β* | *p* |
| --- | --- | --- | --- | --- | --- | --- | --- |
| Auto-Regressive and Cross-Lagged Effects | | |  |  |  |  |  |
| T3 PTRG | ß | Co-reflection – T2 (Cross-lag) |  | 0.17 | 0.06 | 0.18 | <.001 |
|  | ß | PTRG – T2 (Autoregressive) |  | 0.83 | 0.05 | 0.74 | <.001 |
| T3 Co-Reflection | ß | Co-reflection – T2 (Autoregressive) |  | 0.65 | 0.06 | 0.64 | <.001 |
|  | ß | PTRG – T2 (Cross-lag) |  | 0.21 | 0.05 | 0.18 | <.001 |
| *Model without Covariates* | | |  |  |  |  |  |
| Auto-Regressive and Cross-Lagged Effects | | |  |  |  |  |  |
| T3 PTRG | ß | Co-reflection – T2 (Cross-lag) |  | 0.16 | 0.05 | 0.16 | <.001 |
|  | ß | PTRG – T2 (Autoregressive) |  | 0.84 | 0.05 | 0.75 | <.001 |
| T3 Co-Reflection | ß | Co-reflection – T2 (Autoregressive) |  | 0.74 | 0.05 | 0.74 | <.001 |
|  | ß | PTRG – T2 (Cross-lag) |  | 0.22 | 0.05 | 0.19 | <.001 |

#

# **Study 2**

## **Results**

***Factor Analysis of the Post-Transgression Relationship Growth Scale***

We conducted principal components analyses (PCA) on the 32-item static PTRG scale and PTRG scale. Across all three measurements, Bartlett’s Test of Sphericity indicated that the correlation matrix was not random, and the Kaiser–Meyer–Olkin’s (KMO) was above the minimum of .50. Three components emerged from the initial extraction with eigenvalues greater than one (one for T2, and two for T3). Again, like Studies 1 and 2, the first component explained a large amount of the variance (T1: 79.1%; T2: 78.7%; T3: 79.1%). All items loaded strongly onto the first factor. See Supplementary Table 7.

***Exploratory Analyses***

We conducted an exploratory analysis to determine if relationship commitment captured by the item “being committed to each other” would predict relationship partners’ engagement in co-reflection. We used latent true change modeling (Steyer et al., 1997, 2015) to assess whether the baseline commitment would predict the latent change co-reflection from T1-T2. Supplementary Table 8 presents the model fit. Baseline commitment was significantly positively associated with change (increase) in co-reflection, β = 0.38, *SE* = 0.06, *p* < .001.

We conducted exploratory analyses on the outcome variables relating to the hypothetical further wrongdoing presented to participants at T3. We were interested in whether PTRG would be positively associated with a willingness to reconcile with their relationship partner after a further wrongdoing, and the belief that they would engage in co-reflection to address the wrongdoing; and PTRG would be negatively associated with anticipated co-brooding and co-avoidance. We conducted separate regressions for both victims and offenders. PTRG was positively associated with willingness to reconcile, but this was only significant for offenders. Next, PTRG was significantly positively associated with co-reflection for both victims and offenders. PTRG was significantly negatively associated with co-brooding, but only for offenders. Finally, PTRG was not significantly associated with co-avoidance for either victims or offenders. Supplementary Table 8 presents the model fit. Supplementary Table 9 presents the standardized coefficients.

The final consideration was to examine the predictive power of our PTRG scale compared to a change score derived from longitudinal measurement. We opted to use latent true change analyses to compare how our PTRG scale compares as a predictor for these outcomes compared to the latent true change score of the static relationship qualities derived from T1-T2. Supplementary Table 9 presents the standardized coefficients. Both the PTRG, and static RQ true latent change score, were significant positive predictors of willingness to reconcile. However, only the static RQ true latent change score was significant positive predictor of co-reflection. Further, neither the PTRG, nor the static RQ true latent change score significantly predicted co-brooding, or co-avoidance.

**Supplementary Table 7.** The Post-Transgression Relationship Growth Scale and PCA Component Loadings Study 2

| Scale | Loadings (T1, T2, T3) | Scale | Loadings (T1, T2, T3) |
| --- | --- | --- | --- |
| **Mutual Support** |  | **Relationship Flexibility** |  |
| Having compassion for each other. | 0.88, 0.85, 0.86 | Coping with problems that arise in our relationship. | 0.82, 0.88, 0.89 |
| Improving each other’s well-being. | 0.87, 0.89, 0.90 | Changing things that need changing in our relationship. | 0.85, 0.86, 0.85 |
| Supporting each other. | 0.88, 0.92, 0.90 | Finding ways to make things work in our relationship. | 0.89, 0.93, 0.89 |
| Making an effort for each other. | 0.91, 0.90, 0.87 | Trying to make improvements in our relationship. | 0.88, 0.92, 0.85 |
|  |  |  |  |
| **Connection** |  | **Valuing the Relationship** |  |
| Having a sense of closeness. | 0.89, 0.90, 0.91 | Appreciating the value of each other. | 0.89, 0.93, 0.94 |
| Feeling connected. | 0.89, 0.91, 0.88 | Recognizing the importance of each other. | 0.89, 0.93, 0.92 |
| Being committed to each other. | 0.78, 0.89, 0.90 | Valuing being together. | 0.89, 0.93, 0.92 |
| Feeling like one. | 0.80, 0.85, 0.89 | Respecting each other. | 0.85, 0.90, 0.91 |
|  |  |  |  |
| **Open communications** |  | **Trust** |  |
| Expressing our emotions. | 0.78, 0.81, 0.81 | Trusting each other. | 0.85, 0.90, 0.87 |
| Listening to each other. | 0.85, 0.89, 0.89 | Relying on each other. | 0.87, 0.85, 0.92 |
| Communicating openly. | 0.84, 0.86, 0.85 | Believing in each other. | 0.91, 0.91, 0.91 |
| Sharing our thoughts. | 0.85, 0.88, 0.83 | Having faith in each other. | 0.91, 0.92, 0.90 |
|  |  |  |  |
| **New Possibilities within Relationship** |  | **Shared Values** |  |
| Planning the future together. | 0.81, 0.87, 0.89 | Agreeing on what matters in our relationship. | 0.86, 0.88, 0.90 |
| Looking for new things to do together. | 0.80, 0.84, 0.89 | Having a shared sense of what binds us together. | 0.83, 0.87, 0.93 |
| Imagining new possibilities. | 0.81, 0.88, 0.88 | Sharing an understanding of what is important to us. | 0.89, 0.88, 0.93 |
| Setting goals together. | 0.84, 0.90, 0.89 | Being on the same page about what really matters. | 0.81, 0.86, 0.91 |

*Notes.* The T1 + T2 measurement is the static worded scale version.

| Model | χ2 (*df*) | CFI | SRMR | RMSEA [90%CI] |
| --- | --- | --- | --- | --- |
| Baseline Commitment 🡪 ∆ T1-T2 Co-reflection | 100 (40) | .97 | .026 | .091 [.069, .113] |
| ∆ T1-T2 Static RQ + PTRG 🡪 T3 WTR (Vignette) | 79.1 (55) | .99 | .044 | .049 [.021, .072] |
| ∆ T1-T2 Static RQ + PTRG 🡪 T3 Co-Reflection (Vignette) | 44.0 (34) | .99 | .018 | .040 [.001, .071] |
| ∆ T1-T2 Static RQ + PTRG 🡪 T3 Co-Brooding (Vignette) | 56.1 (34) | .99 | .022 | .060 [.029, .087] |
| ∆ T1-T2 Static RQ + PTRG 🡪 T3 Co-Avoidance (Vignette) | 59.4 (34) | .99 | .023 | .064 [.035, .090] |

**Supplementary Table 8.** Model fit statistics

|  | Willingness to Reconcile | |  | Co-Reflection | |  | Co-Brooding | |  | Co-Avoidance | |  |
| --- | --- | --- | --- | --- | --- | --- | --- | --- | --- | --- | --- | --- |
|  | β | *p* |  | β | *p* |  | β | *p* |  | β | *p* |  |
| Regression Analyses    Victims | | |  |  |  |  |  |  |  |  |  |  |
| PTRG | .15 | .19 |  | **.26** | **.03** |  | -.04 | .73 |  | -.07 | .56 |  |
| Offenders |  |  |  |  |  |  |  |  |  |  |  |  |
| PTRG | **.50** | **< .001** |  | **.56** | **< .001** |  | **-.31** | **< .001** |  | -.19 | .05 |  |
|  |  |  |  |  |  |  |  |  |  |  |  |  |
| Latent True Change Analyses | | | | |  |  |  |  |  |  |  |  |
| PTRG | **.19** | **< .001** |  | .13 | .15 |  | -.09 | .31 |  | .09 | .87 |  |
| Static RQ LTC Score | **.22** | **< .001** |  | **.30** | **< .001** |  | -.04 | .64 |  | -.01 | .92 |  |

**Supplementary Table 9.** Standardized coefficients for responses to hypothetical transgression

*Notes*. Statistically significant relationships are bolded. PTRG = post-transgression relationship growth scale; RQ = relationship qualities; LTC = latent true change

# **Supplementary Analyses (8-item PTRG Scale)**

**Table 1.** The Post-Transgression Relationship Growth 8-item Scale with Reported Growth/Decline Across Items (Pilot Study)

| Scale | Experienced Growth | Experienced Decline | Scale | Experienced Growth | Experienced Decline |
| --- | --- | --- | --- | --- | --- |
| **1. Mutual Support** |  |  | **5. Relationship Flexibility** |  |  |
| Supporting each other | 44.8% | 30.2% | Finding ways to make things work in our relationship | 57.6% | 23.3% |
|  |  |  |  |  |  |
| **2. Connection** |  |  | **6. Valuing the Relationship** |  |  |
| Feeling connected | 27.9% | 50.0% | Valuing being together | 51.2% | 25.0% |
|  |  |  |  |  |  |
| **3. Open communications** |  |  | **7. Trust** |  |  |
| Communicating openly | 51.2% | 31.4% | Trusting each other | 23.8% | 59.3% |
|  |  |  |  |  |  |
| **4. New Possibilities within Relationship** | |  | **8. Shared Values** |  |  |
| Imagining new possibilities | 33.1% | 33.7% | Agreeing on what matters in our relationship | 51.2% | 22.7% |

| Model | χ2 (*df*) | CFI | SRMR |
| --- | --- | --- | --- |
| ∆ T1-T2 Static RQ 🡪 8-item PTRG | 539.7 (232) | .94 | .038 |
| ∆ T1-T2 Co-Reflection 🡪 ∆ T1-T2 8-item Static RQ | 667.9 (291) | .94 | .086 |
| ∆ T1-T2 Co-Reflection 🡪 8-item PTRG | 309.8 (131) | .95 | .038 |

**Table 2.** Fit Statistics of Latent True Change Models in Study 2

*Notes.* ∆ = change (delta), RQ = relationship qualities, PTRG = post-transgression relationship growth, all χ2 values are statistically significant.

.17^***^

.65^***^

.19^***^

.82^***^

**Figure 1.** Standardized coefficients between co-reflection and 8-item PTRG (Study 1)

Notes. PTRG = post-transgression relationship growth. The depicted model excludes within-wave correlations of residual variances and T1 covariates.

**.38**

-.07

**.59**

PTRG1

T1

PTRG3

T1

PTRG4

T1

PTRG5

T1

PTRG6

T1

PTRG8

T1

RQ1

T2

T1

RQ1

T1

T1

RQ2

T2

T1

RQ2

T1

T1

PTRG2

T1

RQ3

T2

T1

RQ3

T1

T1

RQ4

T1

T1

RQ4

T2

T1

RQ5

T2

T1

RQ5

T1

T1

PTRG6

T1

RQ6

T2

T1

RQ6

T1

T1

RQ7

T1

T1

RQ7

T2

T1

RQ8

T1

T1

RQ8

T2

T1

**Figure 2.** Standardized coefficients for the structural true latent change modeling of 8-item static RQ to the 8-item PTRG.

Note. RQ = relationship qualities, PTRG = post-transgression relationship growth. Statistically significant relationships at *p* < .001 are bolded. RQ1/PTRG1 = mutual support item etc. (see Table 1). Disturbances, items residuals, and correlations between residuals of matching items across time are omitted from the graph.

**.76**

**.78**

**-.55**

**-.47**

RQ1

T1

T1

RQ2

T1

T1

RQ3

T1

T1

RQ4

T1

T1

RQ5

T1

T1

RQ6

T1

T1

RQ7

T1

T1

RQ8

T1

T1

RQ1

T2

T1

RQ2

T2

T1

RQ3

T2

T1

RQ4

T2

T1

RQ5

T2

T1

RQ6

T2

T1

RQ7

T2

T1

RQ8

T2

T1

CR1

T2

T1

CR1

T1

T1

CR2

T2

T1

CR2

T1

T1

CR3

T2

T1

CR3

T1

T1

CR4

T1

T1

CR4

T2

T1

CR5

T2

T1

CR5

T1

T1

**Figure 3.** Standardized coefficients for the structural true latent change modeling of co-reflection to the true latent change score of the static relationship qualities 8-item scale.

Note. CR = co-reflection, RQ = relationship qualities. Statistically significant relationships at *p* < .001 are bolded. RQ1 = mutual support item etc. (see Table 1). Disturbances, items residuals, and correlations between residuals of matching items across time are omitted from the graph.

**.53**

**-.48**

**.72**

PTRG1

T1

PTRG3

T1

PTRG4

T1

PTRG5

T1

PTRG6

T1

PTRG8

T1

CR1

T2

T1

CR1

T1

T1

CR2

T2

T1

CR2

T1

T1

PTRG2

T1

CR3

T2

T1

CR3

T1

T1

CR4

T1

T1

CR4

T2

T1

CR5

T2

T1

CR5

T1

T1

PTRG6

T1

**Figure 4.** Standardized coefficients for the structural true latent change modeling of co-reflection to 8-item PTRG.

Note. CR = co-reflection, PTRG = post-transgression relationship growth. Statistically significant relationships at *p* < .001 are bolded. PTRG1 = mutual support item etc. (see Table 1). Disturbances, items residuals, and correlations between residuals of matching items across time are omitted from the graph.
